# Estrogen signaling processes in fibroblasts: a scoping review

**DOI:** 10.3389/fendo.2026.1768772

**Published:** 2026-03-19

**Authors:** Neena Roy, Livio Casarini, Darya Smetanina, Shamsa Al Awar, Kornelia Zaręba

**Affiliations:** 1 Department of Obstetrics & Gynecology, College of Medicine and Health Sciences, United Arab Emirates University, Al Ain, United Arab Emirates; 2 Unit of Endocrinology, Department of Biomedical, Metabolic and Neural Sciences, University of Modena and Reggio Emilia, Modena, Italy; 3 Center for Genomic Research, University of Modena and Reggio Emilia, Modena, Italy

**Keywords:** estrogen pathway, estrogen receptor, estrogen signaling, fibroblasts, GPER, phytoestrogen, SERM

## Abstract

Fibroblasts are a heterogeneous cell population with distinct functions, known to respond to estrogens through nuclear estrogen receptor alpha and beta (ERα, ERβ), and the membrane G protein-coupled estrogen receptor (GPER). In this scoping review, we systematically mapped the current evidence on the contribution of estrogen, phytoestrogens, and selective estrogen receptor modulators (SERMs) on estrogen signaling in fibroblasts. A systematic search across PubMed, Scopus and EMBASE was conducted, followed by screening of titles/abstracts, and full text in Covidence, resulting in 67 eligible studies. Our findings reveal that fibroblasts respond to estradiol (E2), phytoestrogens, and SERMs, activating both genomic and non-genomic responses through ERα, ERβ and GPER. These responses contribute to anti-fibrosis, wound healing, anti-inflammatory, and protective effects across diverse fibroblast models. In contrast, in cancer-associated fibroblasts, these ligands can promote cancer in a paracrine manner, emphasizing the role of the tumor microenvironment in cancer progression. However, significant gaps including small sample sizes, high ligand concentrations, lack of mechanistic detail and limited investigation of sex-specific fibroblast responses remain. Addressing these gaps by standardized experimental designs, physiologically relevant models, clearer distinction of receptor-specific pathways and sex-specific analyses in future research will advance the understanding of estrogen-mediated fibroblast signaling and aid in the development of novel therapeutic targets for estrogen-related disorders.

## 1 Introduction

Fibroblasts are specialized cells that regulate tissue homeostasis and repair by synthesizing extracellular matrix (ECM) components and orchestrating signaling microenvironments via biophysical and biochemical stimuli (1, 2). They are a heterogeneous population distributed across diverse organs exhibiting distinct functions and phenotypes. Notably, fibroblasts respond to hormonal signaling, particularly estrogen, through classical estrogen receptor alpha (ERα) and beta (ERβ) in the nucleus (3, 4) as well as the membrane-bound G protein-coupled estrogen receptor (GPER, also known as GPR30) (5–8). The fact that the latter molecule could bind estrogens *in vivo* is under debate (9). Whereas structural and functional data indicated that GPER could not act as a direct estrogen receptor (ER) (10), other reports suggested that the molecule behave as a tissue-specific mediator of estrogen signaling (11). Estrogen signaling occurs via two principal mechanisms: genomic and non-genomic actions. In the genomic signaling pathway, estrogens bind to the cytoplasmic ERα or ERβ, inducing the formation of homo- or heterodimers that translocate to the nucleus, where they interact with estrogen response elements (EREs) on DNA to initiate the transcription of ERE-regulated genes (12–14). While ERα is classically associated with proliferative events, ERβ mediates anti-proliferative effects (14). In the non-genomic pathway, estrogen rapidly signals by binding to membrane-associated ERα or ERβ or to GPER, activating various intracellular signaling cascades that modulate downstream transcription factors and epigenetic regulators (8). These pathways regulate fibroblast activation, proliferation, differentiation, and ECM maintenance during disease (15, 16).

In addition to estrogens, selective estrogen receptor modulators (SERMs) and phytoestrogens can also act through ER to elicit significant biological responses (17, 18). SERMs are structurally diverse, nonsteroidal compounds that exert selective agonistic or antagonistic effects in estrogen-responsive tissues. Widely used examples include tamoxifen, clomifene, chloranifene, raloxifene, ospemifene, lasofoxifene, and bazedoxifene (19). Their effects are influenced by tissue-dependent patterns of ER subtype expression, the composition of co-regulatory proteins, and structural rearrangements of the receptor triggered by ligand engagement (20). SERMs are frequently prescribed for breast malignancies and osteoporotic conditions, where they may elicit different, if not opposite effects, depending on the liganded receptor (21). However, SERMs may also play a role in fibroblast-dependent conditions, such as mitigating skin aging (19) and modulating vaginal atrophy (22). Furthermore, another study reports that tamoxifen may aid in treating keloids by inhibiting fibroblast proliferation (23). It was also reported that tamoxifen induces cytoskeletal remodeling (24) and migration in endometrial cancer cells; this mechanism warrants further investigation in fibroblast tissue due to its potential application in wound healing. Another well-studied SERM, raloxifene, exerts a stronger stimulatory effect on collagen biosynthesis than 17β-estradiol and inhibits matrix metalloproteinase-9 (MMP-9) expression (25). Effects of other SERMs in this field are still under investigation.

Phytoestrogens are structurally distinct, nonsteroidal, plant-derived compounds that display tissue-specific estrogenic or antiestrogenic activities (26). They are characterized by varying degrees of estrogen-like activity and may be proposed as herbal supplements in certain conditions associated with estrogen deficiency (27), although the impact of these molecules on human pathophysiology is debated (28). Depending on the target tissue, phytoestrogens can act as either estrogen agonists or antagonists (29, 30), and they bind with higher affinity to ERβ than ERα (31), with some exceptions (32). Recent studies have demonstrated that phytoestrogens can increase collagen content (33), enhance hyaluronic acid production (34), and promote the synthesis of extracellular matrix proteins (35). The most common phytoestrogens include genistein, daidzein, and resveratrol. Genistein acts as a natural SERM and delays skin aging by increasing dermal collagen thickness through upregulation of subcutaneous vascular endothelial growth factor (VEGF) and transforming growth factor β (TGFβ). It also inhibits matrix metalloproteinases (MMPs) by elevating tissue inhibitor of metalloproteinase (TIMP) levels, thereby reducing collagen degradation (36). Daidzein exhibits a mechanism of action similar to that of endogenous estrogens; it influences the transcriptional activity of extracellular matrix-related genes in dermal fibroblasts and promotes the expression of collagen types I and IV, elastin, and fibrillin‐1 (37). Resveratrol has also been shown to stimulate the production of collagen and elastin while inhibiting MMP activity (38).

Despite the expanding evidence emerging from *in vitro* and *in vivo* studies (39–41), the scope and characteristics of the effects of estrogens, SERMs, and phytoestrogens on fibroblast signaling pathways across tissue types and experimental conditions remain poorly mapped. Accordingly, this scoping review intends to systematically examine the available research regarding these agents’ effects on signaling pathways in fibroblasts across both non-malignant and pathological contexts, providing an overview of the mechanisms and identifying key knowledge gaps. This will improve our understanding of fibroblast biology and inform future studies targeting fibroblast-mediated processes in health and disease.

## 2 Methods

This scoping review was conducted to map the literature on estrogen signaling pathways in the fibroblasts, with a focus on SERMs and phytoestrogens. Our methodology has been developed in accordance with the Joanna Briggs Institute (JBI) methodology for scoping reviews (42) and has been reported following the recommendations for the Preferred Reporting Items for Systematic Reviews and Meta-analyses extension for Scoping Reviews (PRISMA-ScR) (43). The protocol was registered with the Open Science Framework on October 15, 2025 (44).

### 2.1 Search strategy

We performed a comprehensive literature search across three databases: PubMed (RRID: SCR_004846), Scopus (RRID: SCR_022559) and EMBASE (RRID: SCR_001650). Our search terms included controlled vocabulary and free-text words relating to “fibroblast”, “estrogens”, “SERMs”, “phytoestrogens”, “signaling pathway”, “*in vitro*” and “*in vivo*”. Boolean terms, AND and OR, were used for the search. Medical Subject Headings (MeSH) terms for keywords were also included. The search string was tailored to suit each database. Full search string for each database is provided in **Supplementary Table 1**.

The literature search commenced on July 15, 2025, and aimed to capture all relevant articles up to July 2026, including those available online ahead of print.

### 2.2 Eligibility criteria

Eligibility criteria were designed to capture experimental studies directly examining estrogen, SERM, or phytoestrogen-mediated signaling pathways in fibroblasts, while excluding studies without fibroblast-specific mechanistic data. This review was conducted following the Population–Concept–Context (PCC) framework, which guided both the search strategy and study selection process. Eligible studies investigated fibroblasts from any tissue origin and examined estrogen-, SERM-, or phytoestrogen-mediated intracellular signaling pathways, including those involving ERα, ERβ, and GPER. Studies employing *in vitro* (primary fibroblasts or established cell lines) or *in vivo* (when fibroblast-specific signaling responses were explicitly examined or characterized) experimental models were included. Only original experimental research articles published in peer-reviewed journals in English between January 2015 and July 2026 were considered.

Studies were excluded if they: (i) focused on non-fibroblast cell types; (ii) did not report estrogen-, SERM-, or phytoestrogen-mediated signaling pathways; (iii) were clinical, epidemiological, or computational investigations lacking experimental fibroblast models; (iv) involved *in vivo* models where fibroblasts were not explicitly examined or outcomes could not be specifically attributed to fibroblast signaling; or (v) were reviews, editorials, commentaries, conference abstracts, preprints, grey literature, retracted publications, or non-English manuscripts. Computational studies were excluded because the primary aim of this scoping review was to map experimentally validated ER mediated signaling pathways in fibroblasts, and inclusion of purely computational studies would have limited comparability across mechanistic outcomes.

### 2.3 Selection of sources of evidence

All retrieved records were uploaded to the web application Covidence (RRID: SCR_016484) (45) for screening, and duplicates were automatically removed. Two reviewers (NR and KZ) independently assessed titles and abstracts and resolved any conflicts through discussion. Duplicates identified at this stage were removed manually. Articles passing the initial screening underwent full-text review by the same two reviewers independently, with disagreements resolved by consensus. Both screening stages adhered to the inclusion and exclusion criteria. This independent dual-reviewer screening process was implemented to ensure consistency in study selection and to minimize potential selection bias.

### 2.4 Data charting and synthesis of results

Data extraction was performed by one reviewer and independently verified by a second reviewer for accuracy and completeness. Any discrepancies were resolved through discussion and consensus. Data was extracted using a data extraction form prepared in Microsoft Excel (Microsoft Office LTSC Professional Plus 2021, RRID: SCR_016137). From each included study, we extracted: first author and year of publication, tissue of fibroblast origin, species, ligand/modulator used, dose/concentration/treatment exposure, receptor/signaling pathway studied, main findings/relevance related to the scoping review, and study limitations. Under limitations, we extracted both the author-reported relevant limitations as well as additional limitations identified by us during the review process.

The extracted data were tabulated and categorized into five mechanistic groups (1), estradiol (E2) acting via ERs only (2), E2/G1 (GPER agonist)/G15 (GPER antagonist) signaling through GPER only (3), E2 acting via both ERs and GPER (4), phytoestrogen-mediated signaling, and (5) SERM-mediated signaling, based on the ligands, modulators and receptors reported in each study. Within each group, studies were further examined for specific attributes, including anti-fibrotic, pro-fibrotic, pro-proliferative, anti-apoptotic, and anti-inflammatory effects, which were subsequently analyzed and discussed to elucidate overarching trends and mechanistic insights.

### 2.5 Quality Assessment

A formal assessment of risk of bias or methodological quality was not conducted, as this is not mandatory for scoping reviews (42). However, the quality of the included sources was appraised using bibliometric indicators, including the h5-index, journal impact factor, and Scimago Journal Rank (SJR)/quartile classification, to provide contextual information on the credibility and influence of the evidence base (**Supplementary Table 2**).

## 3 Results

### 3.1 Study selection and characteristics

**Figure 1:**
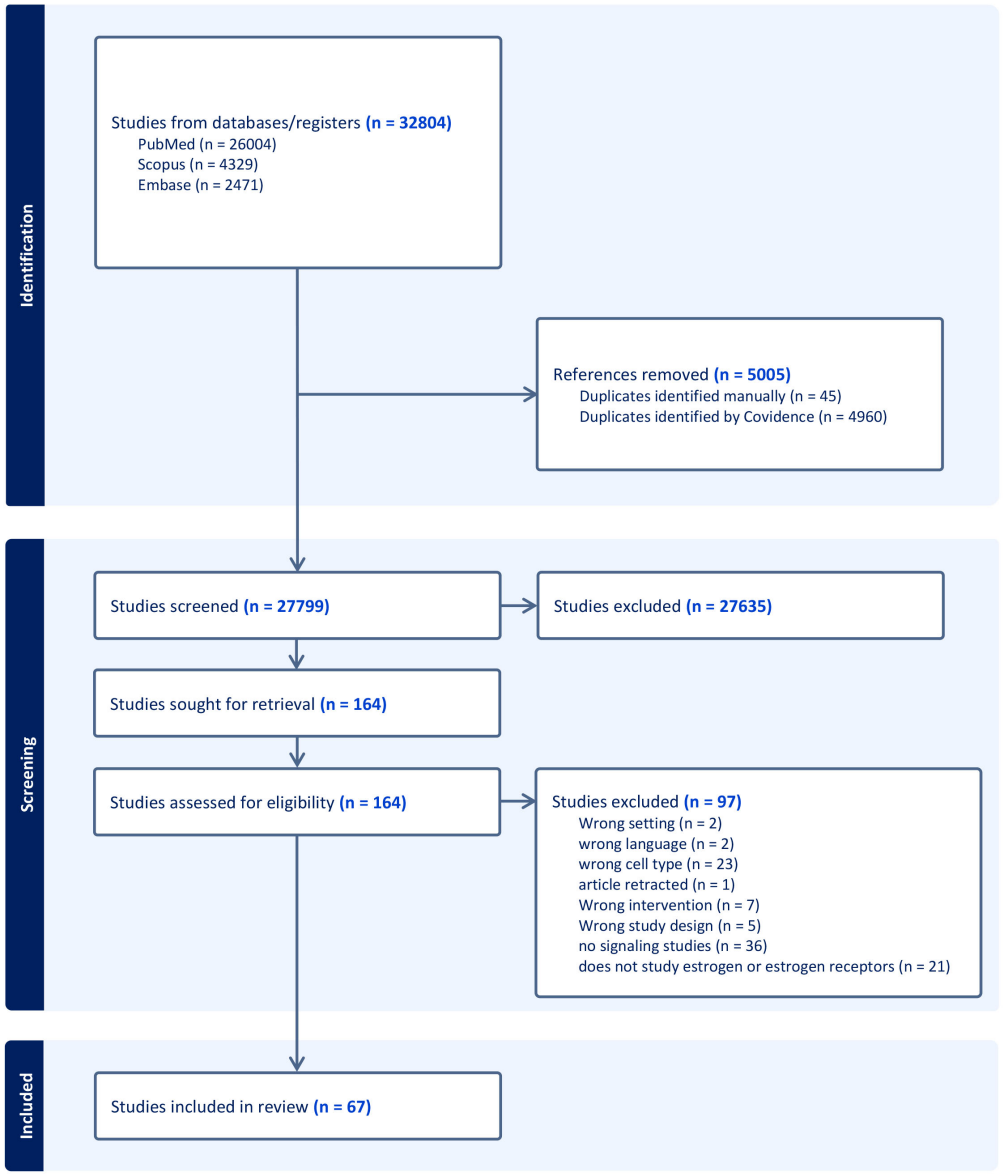
Flowchart presenting the PRISMA process for study selection.

Our literature search retrieved 32,804 records: 26,004 from PubMed, 4,329 from Scopus, and 2,471 from Embase. After identifying duplicates (4,960 detected by Covidence and 45 removed manually), 27,799 were screened by title and abstract. Of these, 164 studies proceeded to full-text screening, resulting in 67 studies meeting the eligibility criteria for data extraction (**Figure 1**).

Among the 67 included studies, 32 investigated ER-dependent genomic responses to E2 (**Table 1**), 17 focused on GPER-specific mechanisms (**Table 2**), 3 examined combined ER and GPER involvement (**Table 3**), 10 assessed phytoestrogens (**Table 4**), and 5 evaluated SERMs (**Table 5**). The fibroblasts studied were either primary or immortalized cell lines derived from human, mouse, or rat sources. Tissue origins included cardiac, uterine, mammary, dermal, Tenon’s capsule, tunica albuginea, vocal fold, sub-synovial connective tissue, ovarian, uterosacral ligament, nipple, keloid, synovial, lung, and embryonic tissues, as well as cancer-associated fibroblasts (CAFs) derived from breast, prostate, and gastric tumors.

**Table 1:** Characteristics of the classical ER-dependent signaling in fibroblasts.

Author/ Year	Fibroblast origin	Species	Ligand/ Modulator	Dose/concentration/ treatment exposure	Signaling Pathway/outcome	Relevance	Limitations
Avouac et al., 2020 (46)	Dermal fibroblasts from systemic sclerosis patients	Human	E2	Recombinant TGFβ: 10 ng/mL, 24 h. Tamoxifen: (5 μg/mL; E2: 10 μg/mL, 24 h; MG132 (proteasome inhibitor: 10 or 20 μl); MPP (ERα inhibitor: 10^-6^ M); PHTPP (ERβ inhibitor: 10^-6^ M)	E2 → ERα and ERβ → proteasome dependent degradation of pSMAD2/3 → ↓ SMAD signaling → ↓ collagen, ↓ αSMA → anti-fibrotic	Anti-fibrotic role of E2 in TGFβ induced systemic sclerosis.	The study did not use male fibroblasts or postmenopausal fibroblasts, however, the findings were extrapolated to fibrosis severity observed in men and post-menopausal women with reduced E2 levels.
Ling et al., 2025 (47)	Colon fibroblasts (CCD-18Co)	Human	ERB041 (ERβ agonist)	Stimulation: TGFβ1: 10 ng/mL, 2 h. Treatment: ERB041: 100 nM, 46 h following TGFβ exposure	ERB041 → ERβ activation → (↓ TGFβ/Smad signaling: TGFβ, p-Smad2, p-Smad2/3, TGFβ receptor II (TGFβ RII) and ↓ TLR4/MyD88/NF-κB → inhibition of the nuclear translocation of p65) → ↓ fibroblast activation and ↓ α-SMA, vimentin, collagen I, and N-cadherin	ERβ activation promotes anti-fibrotic effects in intestinal fibroblasts.	No primary fibroblasts were used.
Ahluwalia et al., 2022 (48)	Cardiac fibroblasts	Mice	E2	AngII (100 nM) ± E2 (10 nM) / β-LGND2 (ERβ agonist: 10 nM), 24 h	E2 → ERβ → inhibits AngII mediated phosphorylation and nuclear translocation of SMAD3 → blocks fibroblast to myofibroblast transition → anti-fibrotic	E2 mitigates cardiac cell pathologies through membrane associated ERβ.	Mechanistic link between ERβ and SMAD3 is lacking.
Pedram et al., 2016 (49)	Cardiac fibroblasts	Sprague-Dawley rats	E2	AngII or ET-1 (100 nM), ± E2 (10 nM), or βLGND2 (10 nM), or DPN (10 nM), 24 h. TGFβ1 (10 ng/mL, 1 or 16 h)	E2 → ERβ → cAMP/PKA and AMPK → ↑ phosphorylation of RhoA at Ser188 (inactive form) → ↓ Rho kinase activity → ↓ TGFβ1 and connective tissue growth factor (CTGF) expression → ↓ MMP2 and MMP9 expression and activity, and the conversion of fibroblasts to myofibroblasts → Collagen I and II decreased	E2/ERβ signaling prevents Ang II and ET-1-induced cardiac fibrosis.	The study used neonatal rat fibroblasts, therefore, the findings may not translate to adult cardiac fibroblast physiology or human cardiac remodeling due to developmental and species-specific differences.
Yamanaka et al., 2018 (50)	Fibroblasts from subsynovial connective tissue of postmenopausal idiopathic carpel tunnel syndrome patients	Human	E2	E2: 10^−4^ –10^−12^ M, 24 h	E2 → ERα activation → ↓ TGFβ–responsive gene transcription → ↓ Col1A1 and Col3A1 mRNA → ↓ collagen I and III protein → ↓ fibroblast-driven fibrosis	E2’s anti-fibrotic mechanism alleviates idiopathic carpel tunnel syndrome-associated pathologies.	Only postmenopausal women were studied; premenopausal women and men were not included. A supraphysiologic dose of E2 was used, and downstream estrogen receptor (ER) signaling was not explored.
Jiang et al., 2015 (51)	Tunica albuginea fibroblasts	Sprague-Dawley rats	E2	E2: 10 nM ± TGF: 5 ng/mL, 24 h	E2 → inhibits TGFβ mediated signaling (↓ Smad2 phosphorylation and RhoA-ROCK2 expression) → ↓ collagen and ↓ myofibroblast contraction	Estrogen’s protective role in Peyronie’s disease via suppression of TGFβ1–mediated fibrotic signaling.	ER subtype involvement (ERα, ERβ, or GPER) was not defined. Did not explore long-term exposure. Species-specific findings may not fully translate to human fibroblast biology.
Yang et al., 2023 (52)	Tenon fibroblasts	Human	E2	TGFβ (5 ng/mL, 60 min) + E2 (12.5- 100 μmol/L), 24 h or 3 days	E2 → ↓ TGFβ–induced Smad2/3, MAPK (p38 and ERK) phosphorylation → ↓ MMP-1/3, MCP-1, IL-6, and VEGF expression → ↓ collagen gel contraction, stress fiber formation	Anti- inflammatory and anti-fibrotic effects of E2 and its significance in preventing scar development and inflammation in the conjunctiva.	Effective inhibition required ≥ 25 µM, a supraphysiologic concentration of E2, and the specific ER involvement was not defined.
Xu et al., 2024 (53)	Immortalized cardiac fibroblasts	Mice	E2	E2: 5, 10, and 50 nM (added after 6 h of TGFβ1 (20 ng/mL) pre–treatment), continued 48 h	E2 → ↑ Cdc42→ ↑ Pak1, JNK and c-Jun → ↓ TGFβ1 → ↓ α-SMA, collagen I and collagen III	E2 ameliorates myocardial fibrosis through a Cdc42-dependent pathway.	Primary fibroblasts were not used, and ER involvement was not explored.
Darawsha et al., 2021 (54)	Dermal fibroblasts	Human	E2	E2 (10 nM), medium replaced ± H_2_O_2_ (25 μM/ 50 μM, 90 min/ 12 h/ 24 h)	E2 → increase in ARE/Nrf2 transcription system → increase in anti-oxidant enzymes → reduced ROS. Tomato and rosemary extracts, E2 → ↑ MMP1 and ↓ pro-collagen → cell damage protection	Protective effect of estradiol under oxidative stress.	The mechanistic link between antioxidant pathway activation and the observed reduction in MMP-1 and increase in pro-collagen is incomplete. Specific ER involved was not explored.
Darawsha et al., 2024 (55)	Dermal fibroblasts	Human	E2	E2 (10 nM), 24 h and medium replaced ± rotenone (1 μM, 90 min/ 4 h/ 48 h)	E2 → increase in ARE/Nrf2 transcription system → ↑ NQO1 (anti-oxidant enzyme) → reduced ROS → ↑ MMP1 and ↓ pro-collagen → cell damage protection	Protective effects of estradiol from mitochondrial oxidative stress and their importance in delaying skin ageing.	Cross-talk with other pathways was not examined, and the persistence of antioxidant effects under chronic stress remains unknown. The mechanistic link between Nrf2 and ROS is incomplete, and estradiol signaling was not clearly defined.
Bakerfrost et al., 2021 (56)	Dermal fibroblasts	Human	E2	Pre–treatment for 1 h with inhibitors (U0126: 10 μM; fulvestrant: 100 nM; SB-431542: 10 μM) ± E2, 24 h/ 48 h	E2 → ERα → MAPK activation → ↑ early growth response 1 (EGR1) → ↑ TGFβ1 and TGFβ2	Pro-fibrotic effects of E2 in dermal fibrosis.	Only healthy dermal fibroblasts were tested. Diseased fibroblasts were not directly compared under the same E2 exposure.
Bakerfrost et al., 2024 (57)	Dermal fibroblasts from systemic sclerosis patients	Human	E2	E2 (10 nM), recombinant IL-6 (20 ng/mL), 24 or 48 h	(E2 → ERα → ↑IL-6 → ↑ aromatase activity → ↑ E2 → ERα → ↑IL-6) → this loop activates pro-fibrotic mediators (Col IIIA1, Col VA1, and fibronectin) → dermal fibrosis	Pro-fibrotic effect of IL-6 in skin and positive feedback interaction between IL-6 and E2.	Intracellular pathway downstream of ERα is not mapped.
Dworatzek et al., 2019 (58)	Cardiac fibroblasts (CFs)	Wistar rats and human	E2	E2 (10^-8^ M, 1 h/12/24 h), ERα agonist: 4,4',4''-(4-propyl-[1H]-pyrazole-1,3,5-triyl) trisphenol (PPT), ERβ agonist (Karo Bio): (10^-7^ M, 24 h)	Female CFs: E2 → serine 118 phosphorylation of ERα → forms heterodimer with ERβ → nuclear translocation and binding to ERE within collagen I and III promoter in presence of Co-repressor → downregulation of collagen I and III genes. Male CFs: E2 → ERβ phosphorylation at serine 105 → ERβ homodimer → nuclear translocation and binding to ERE within collagen I and III promoter in presence of Co-activator → upregulation of collagen I and III genes	Sex specific regulation of E2 and ER signaling in cardiac fibrosis.	Although E2 produced sex-opposite effects on collagen I and III in human fibroblasts, the mechanistic insights were derived solely from rats, with human relevance inferred from conserved Col1A1/Col3A1 promoter sequences rather than direct evidence of ER signaling in human cells.
Ouyang et al., 2016 (59)	Breast cancer associated fibroblasts (CAFs)	Human	E2	E2: 10 μM, 48 h	E2 → ↑ SDF-1α (secreted by CAFs) → recruits MDSCs (myeloid-derived suppressor cells) via CXCR4 → suppression of immune activity and promotes tumor progression	Tumor-promoting role of estrogen in ER-negative breast cancer.	The ER involved was not defined and a supraphysiological dose of E2 was used.
Bae et al., 2022 (60)	Gastric cancer associated fibroblasts (CAFs) lines	Human	E2	E2: 0-10 nM, 24 h/72 h	E2 → ERα in CAF → ↑ CD147 → ↑ MMP2/ MMP9 in CAFs and cancer cells → epithelial- mesenchymal transition → ↑cancer migration and invasion	Estrogen signaling activation in CAFs promotes cancer progression in paracrine manner through CD147 by cancer cells.	Number of CAF lines tested was limited and incomplete mapping of downstream signaling.
Hu et al., 2024 (61)	Fibroblasts from ovarian metastases	Human	E2	E2: 0, 50, 100 nM for 24 h. Fulvestrant: 100 nM for 24 h (± E2). Transwell co-culture setup: Ovarian fibroblasts pre-treated 24 h with E2 (100 nM), Fulvestrant (100 nM), or iMDK (100 nM); medium then replaced and gastric cancer cells added; 72 h	E2 → ERs → ↑ MDK secretion → binds LRP1 on tumor cells → proliferative and metastatic signals	An estrogen-driven ER–MDK–LRP1 signaling axis in fibroblasts promotes ovarian metastasis in gastric cancer, highlighting the role of female hormonal influence in metastatic progression.	Small sample size, underlying mechanism by which ovarian fibroblasts promote MDK secretion in response to estrogen stimulation needs to be explored.
Morgan et al., 2018 (62)	Organotypic mammary duct model (collagen-embedded lumen lined with MCF7 breast cancer cells (ER^+^), surrounded by matrix with or without mammary fibroblasts	Human	E2	E2: 100 nM, 24–72 h	Fibroblast + MCF7 cells → E2 → ↑ ER transactivation → ↑ proliferation and ↓ apoptosis → ductal hyperplasia	Stromal fibroblasts modulate estrogen signaling in ER^+^ breast epithelial cells through paracrine, non–ER-dependent mechanisms, and the study demonstrates the utility of an organotypic 3D duct model for investigating stromal–epithelial interactions.	Non- ER fibroblast mechanisms were not characterized. High dose of E2 was used and the model is less throughput than traditional *in vitro* systems, due to the incorporation of the biomimetic ductal structure, matrix proteins and additional cell types.
Xie et al., 2022 (63)	Uterosacral ligament fibroblasts from pelvic organ prolapse patients	Human	E2	E2: 10^−10^ M and 10^−9^ M and mechanical stress was applied for 24 h	E2 → ERα → ↑PARP1 → ERα transactivation → ↑ Bcl-2 and ↓ Bax expression → anti-apoptosis	Estrogen ameliorates mechanical stress induced cell apoptosis and death.	Small sample size and short-term fibroblast culture; mechanistic validation of PARP1–ERα signaling lacking, and estrogen’s short half-life may limit its observed protective effect.
Wu et al., 2017 (64)	Nipple fibroblasts	K14-PTHrP (KrP) transgenic mouse line	E2	E2: 0.5 nM and 10 nM, 48–72 h	E2 → ERα activation → ↓ Tgfb1 expression → ↓ pSmad2/3 signaling → reduced fibroblast-derived inhibition of epidermal proliferation → maintenance of thick nipple epidermis	Estrogen, via ERα in fibroblasts, represses TGFβ signaling to maintain specialized nipple epidermis.	Mechanistic detail is lacking regarding downstream signaling pathways mediating the effects of E2 in fibroblasts.
Zhang et al., 2023 (65)	Keloid fibroblasts	Human	2-methoxyestradiol (2ME2)	2ME2: 6.975 μM (IC50) ± doramapimod (p38 inhibitor): 20 μM, 48- 96 h	2ME2 → ↓ p38 → ↓ c-Myc / ATF2 / c-Jun → ↓ PCNA and HIF-1α → ↓ keloid fibroblast proliferation	2ME2 exhibits antiproliferative and pro-apoptotic effects in keloid fibroblasts and can be used in keloid management.	Small patient-derived fibroblast sample was used, and the specific ER involved was not delineated.
Zhang et al., 2018 (66)	Keloid fibroblasts	Human	2-methoxyestradiol (2ME2)	2ME2: 6.975 μM (IC_50_) ± Ac-DEVD-CHO (caspase inhibitor): 8 μM, 48 h	2ME2 → ↑ Caspase-8/9/3 → Bax activation → ↓ Bcl-2/Bax ratio → ↑ Cytochrome-c release → ↑ Apoptosis → ↓ PCNA → ↓ Fibroblast proliferation	2ME2 induces apoptosis via caspase activation and reduces fibroblast proliferation, providing a potential targeted therapy for keloid fibrosis.	No exploration of upstream ER involvement and small sample size.
Malik et al., 2024 (67)	Synovial fibroblasts from rheumatoid arthritis patients	Human	E2	E2: 1 μM, 24 h	E2 → ↑ 1- methyl nicotinamide → ↓ STAT, MMP3, MMP9 and MAPK14 → ↓ inflammation and oxidative stress	E2 mitigates rheumatoid arthritis pathogenesis.	No receptor mechanism was explored.
Santos et al., 2017 (68)	Embryonic fibroblasts	Mice C57BL/6J	17α-estradiol (17α-E2) and 17β-estradiol (17β-E2)	17α-E2 and 17β-E2: 10 μM; pre-incubation 1h or 12 h + Lipopolysaccharide (LPS): 10 ng/mL, 2 h	17α-E2 / 17β-E2 → ERα activation → ↓ NFκB-p65, ↓ Tnf-α, ↓ Il-6, ↑ Il-4, ↑ Il-6ra → anti-inflammatory	Anti-inflammatory role of 17α-E2 and 17β-E2 in fibroblasts.	A high (pharmacological) estradiol concentration (10 μM) was used, which may not reflect physiological exposure levels, and the study relied on embryonic fibroblasts, limiting translation to adult tissue physiology.
Song et al., 2019 (69)	Embryonic fibroblasts	Mice C57BL6/129SV	E2	E2 (10 nM, 48 h) ± TNF-α (10 ng/mL, 6 h) ± PHTPP (ERβ antagonist) (10 µM, 48 h)	E2 → Nrf2 → binds to antioxidant response elements (AREs) in ERβ promoter → ERβ expression restored → NF-κB inhibition → ↓ iNOS → inhibits TNF- α mediated inflammation	Nrf2 is pivotal for estrogen’s anti-inflammatory effects in embryonic fibroblasts.	*In silico* analysis was used to predict Nrf2 binding to the ERβ promoter; however, actual binding was not demonstrated.
Cheng et al., 2025 (70)	Fibroblasts from Pelvic Organ Prolapse (POP) patients	Human	E2	E2: 10^−7^ mol/L, 48 h	E2 → SIRT-1/p53/p21 pathway → Inhibition of POP	Anti-senescence effect of E2 and importance of E2 on POP treatment.	Other signaling pathway on how E2 exerts anti-aging effects in POP-derived human fibroblasts and the specific receptor involved were not investigated.
Ma et al., 2021 (71)	Cardiac fibroblasts	Sprague–Dawley rats	17-E2-NPs (nanoparticles)	Myocardial infarction induced rats were injected with 17-E2-NPs (4 mg/100 g) at the infarct border zone	17-E2-NP → ↓ miR-302b expression → ↓ proliferation and ↑ apoptosis	E2 regulates miR-302b involved in myocardial infarction.	Fibroblasts were not directly treated with E2 nanoparticles, and downstream signaling pathways were not dissected. The study did not characterize the receptor involved.
Feng et al., 2019 (72)	Synovial fibroblasts from Idiopathic Condylar Resorption (ICR) Patients	Human	E2	E2: 0.1-10 ng/mL, 12 h; E2: 10 nmol/L, 24 h; miRNA-101-3p mimics (50 mmol/L), inhibitor (100 mmol/L), short interfering RNA (50 mmol/L)	E2 → ↑ miRNA-101-3p expression → ↓ HAS2 expression by direct binding to its 3'-untranslated region	Pathogenesis of ICR.	Small patient cohort and the study focused on a single pathway in a multifactorial disease like ICR.
Midgley et al., 2016 (73)	Young and aged fibroblasts from lungs and dermis	Human	E2	E2: 10^-7^M ± TGFβ /IFNγ: 10 ng/mL; 24 h/72 h	E2 → ER → ↓ STAT1 expression and activity and attenuated binding of STAT1 to the miR-7 Promoter → ↓ miR-7 expression and ↑ EGFR. E2 restored TGFβ1-induced α-SMA, HAS2, and EDA-FN expression in aged fibroblasts	E2 restored aged fibroblasts to a ‘young state’ in preparation for TGFβ1-driven differentiation and thus, E2 has a protective effect on chronic wound healing in the elderly.	Did not assess other aging- or estrogen-related signaling pathways.
Almuntashiri et al., 2024 (74)	Lung fibroblasts (IMR90)	Human	E2	E2: 100 nM, 6 h /24 h	E2 → ERα → binds TIMP-1 promoter → increases TIMP-1 expression	Sex-specific increase in TIMP-1 serves as a biomarker in female patients with SARS-CoV2- and Influenza A virus-related acute lung injury.	Signaling link between ERα and TIMP-1 is incomplete.
Patel et al., 2018 (75)	Fibroblasts from endometrium (EM)	Human	E2	E2: 5×10⁻⁸ to 5×10⁻¹⁰ M, 48 h pre-treatment, then 24 h with/without stimuli. Raloxifene (ERα antagonist: 5×10⁻⁶ M); Poly(I:C) (viral mimic: 0.25–25 µg/mL), 24 h; IFNβ (1–100 U/mL); α-IFNAR2 (10 µg/mL); α-TLR3 (20 µg/mL)	E2 → ↓ Poly(I:C)-induced MxA and OAS2. E2 → ERα → ↑ SDF-1α. E2-treated EM fibroblasts reduced IIIB infection of CD4+ T cells → SDF-1α/CXCR4 interference	Endometrial fibroblasts are responsive to E2, initiate antiviral interferon-mediated response against viral pathogens, and may help prevent HIV infection of CD4+ T cells.	Small donor cohort and incomplete understanding of the mechanism underlying E2-mediated interferon-stimulated gene suppression and downstream ER signaling in EM fibroblasts.
Patel et al., 2018 (75)	Uterine stromal fibroblasts	Human	E2	E2: 5×10⁻⁸ M (48 h pre-treatment); Raloxifene (ERα antagonist: 5×10⁻⁶ M); Poly(I:C) (viral mimic: 0.25–25 µg/mL, 24–48 h; Recombinant IL-27 (1–100 ng/mL), 24–48 h	E2 + Poly(I:C) → ↑ IL-27 and E2 → ERα → ↓ IL-27 induced indoleamine 2,3-dioxygenase (IDO)	Menstrual cycle-dependent IL-27 production in response to pathogens, particularly when the concentration of E2 is high, such as mid-cycle and mid-secretory phases.	Downstream ERα–IL-27 signaling pathways were not characterized, and the use of a single hormone dose with short-term exposure does not reflect physiological E2 fluctuation.
Patel et al., 2021 (76)	Uterine fibroblasts	Human	E2	E2: 5 × 10⁻⁸ M, 48 h followed by 24 h co- exposure with IFNλ1 (500 ng/mL) or Poly(I:C) (viral mimic: 25 µg/mL)	E2 → no significant effect on IFNλ1-induced interferon–stimulated gene expression in fibroblasts	Uterine fibroblasts responded weakly to IFNλ1 and were insensitive to E2, indicating limited ER–dependent control of their antiviral pathway.	The mechanism of E2 insensitivity is undefined, and a small fibroblast sample size was used.

This table summarizes experimental studies investigating ER–dependent (ERα and/or ERβ) signaling pathways in fibroblasts. Information includes fibroblast tissue origin, species, ligand or modulator used, dose and exposure conditions, downstream signaling pathways, reported biological effects, and study-specific limitations.

**Table 2:** Characteristics of GPER signaling in fibroblasts.

Author/ Year	Fibroblast origin	Species	Ligand/ Modulator	Dose/concentration/ treatment exposure	Signaling Pathway/outcome	Relevance	Limitations
Wang et al., 2019 (77)	Cardiac fibroblasts	Mice	G1	CFs were treated for hypoxia/ serum deprivation (H/SD) 12 h, H/SD (12 h) + G1 (10 nM), H/SD (12 h) + Wortmanin (PI3K inhibitor, 100 nM), and H/SD (12 h) + G1 + Wortmanin.	G1 → GPER → activated PI3K/AKT → ↓ BAX, ↓ Caspase-3, ↑ BCL-2 → ↓ apoptosis	Myocardial protective effect of GPER.	Fibroblast strain was not specified, limiting reproducibility, and the upstream or downstream intermediates of the PI3K/AKT pathway were not explored.
Carnesecchi et al., 2015 (78)	Dermal fibroblasts	Human	E2, G1, G15	E2 (10^-8^ M, 24 h) or G1(10^-8^ M) ± G15 (10^-7^ M) or PD98059 (ERK inhibitor: 10^-5^ M)	E2 → GPER → ERK1/2 → cytoskeleton remodelling	Protective effects of E2 on skin through GPER.	Effectors downstream of ERK1/2 was not investigated. ER levels in fibroblasts may vary with anatomical site or the donor’s hormonal state. This variability could influence how fibroblasts respond to estrogen.
Wang et al., 2021 (79)	Neonatal cardiac fibroblasts co-cultured with cardiomyocytes	Sprague–Dawley rats	G1, G15	Cardiomyocytes: Dox (0.1 μM, 24 h); Dox + Ang II (0.1 μM each, 24 h) ± G1 (10 nM, 24 h) or G15 (1 μM, 24 h) or PD98059 (10 μM, 24 h); the conditioned medium transferred to cardiac fibroblasts for 24 h	G1 → GPER → inhibition of ERK1/2 → ↓ MMP9 (cardiomyocytes) → ↓ TGFβ expression (fibroblasts) → ↓ myocardial fibrosis and preserving cardiac function	GPER activation protects against cardiac pathological hypertrophy in postmenopausal females and demonstrates cross-talk between cardiomyocytes and fibroblasts through ERK1/2–MMP-9–TGFβ1 signaling.	Fibroblast activation was examined indirectly using cardiomyocyte-conditioned media rather than direct GPER stimulation, and GPER knockout or transgenic models were not employed to confirm mechanistic specificity.
Wang et al., 2025 (80)	Synovial fibroblasts from frozen shoulder	Human	E2, G1	E2: 10 nM ± G15 (2.5/10 μM), 24 h; G1: 1 μM ± G15 (1/10 μM), 24 h; G1+ MK-2206 (Akt inhibitor)	E2 / G1 → ↑ GPER protein and activation → ↓ PI3K and pAkt → ↓ FN, COL1, COL3, α-SMA → reduced fibroblast activation and collagen deposition → anti-fibrotic effect	E2 exerts anti-fibrotic effects through GPER-mediated PI3K/Akt inhibition in fibroblasts and physiologic/ pathologic estrogen withdrawal is identified as a risk factor for frozen shoulder development.	The concentration of MK-2206 was not reported, which limits reproducibility of the PI3K/Akt inhibition experiments, and the small number of patient-derived fibroblast samples further restricts generalizability.
Liu et al., 2022 (81)	Cardiac fibroblasts	Sprague–Dawley rats	G1	Concentration not mentioned	G1 → GPER → ↓ TGFβ1 → ↓ Smad2/3 phosphorylation → ↑ Smad 7 expression → inhibited Angiotensin II induced α- SMA	GPER alleviates post-menopause related atrial fibrosis.	*In vitro* dose was not stated. Anti-fibrotic effects of G1 were not validated by using a GPER antagonist or GPER knockdown.
Wang et al., 2015 (82)	Cardiac fibroblasts	Sprague-Dawley rats	G1	G1: 10⁻¹⁰–10⁻⁵ M, 24 h	G1 → GPER → ↓ CDK1 and cyclin B1 → ↓ proliferation → ↓ α-SMA → ↑ MMP-12 and ↓ TIMP-1	Protective role of GPER against cardiac fibrosis.	Cultured fibroblasts may undergo phenotypic changes with passaging, potentially altering their response to GPER activation. Only male cardiac fibroblasts were examined, leaving possible sex-related differences unaddressed, and the downstream pathways linking GPER activation to CDK1/cyclin B1 suppression remain incompletely defined.
Wang et al., 2018 (83)	Cardiac fibroblasts	Sprague-Dawley rats	G1	G1 (10 nM), G15 (1 μM), and W1400 (iNOS inhibitor: 1 μM), 48 h	G1 → GPER → ↓ iNOS expression → ↓ NO → ↓ proliferation → ↓ fibrosis	Activation of GPER inhibits myocardial fibrosis.	Other key mediators, such as TNF-a, IL-1b, LDH, ROS levels, p-c-jun, BAX, CTGF, iNOS, and COX2, were not analyzed.
DeMarco et al., 2016 (84)	Breast cancer associated fibroblasts (CAFs)	Human	E2, G1	E2 (10 nM, 4-24 h), G1 (100 nM, 4-24 h)	CAFs (E2/G1 → GPER → EGFR/ERK/PKC → IL1β) → breast cancer cells (E2/G1 → GPER → EGFR/ERK/PKC → IL1R1/IL1β → ↑ PTGES, COX-2, RAGE, ABCG2) → invasion and migration	GPER-mediated estrogen signaling links the tumor microenvironment with tumor cells through a feed-forward loop to promote breast cancer.	CAFs were isolated from only six breast cancer patients, providing proof-of-concept but limited representation of patient variability.
Luo et al., 2016 (85)	Breast cancer associated fibroblasts (CAFs)	Human	E2, G1, G15	E2: 100 nM; G1: 1 μM; G15: 1 μM; Ca^2+^: E2/G1/G15, 150 s; ERK phosphorylation: E2/G1, 15 min ± G15 pre-treatment; Proliferation: E2/G1, 72 h ± G15; Migration: E2/G1, 24 h ± G15; Adhesion: E2/G1, 20 h ± G15	E2/ G1 → GPER- mediated signaling (rapid actions) → ↑ Ca²⁺ and ERK1/2 phosphorylation; slow actions → ↑ proliferation, adhesion, spreading, migration	Estrogen promotes breast cancer via GPER-mediated and CAF-dependent manner.	Small sample size, absence of fibroblast–tumor interaction models, and narrow mechanistic scope confined to the Ca²⁺ /ERK1/2 axis.
Jia et al., 2016 (86)	Prostate cancer associated fibroblasts (CAFs)	Human	GPER was modulated by overexpressing it or siRNA knockdown	Forskolin (1 µM, 24 h) or H89 (2 µM, 72 h), GPER and ER activity (genetic overexpression or siRNA knockdown, 72 h)	GPER → ↓ cAMP → ↓ PKA → ↓ ERα expression → ↓ smooth muscle differentiation markers and ↑ activated CAF markers (SMemb/CD44/FAP)	GPER acts as a negative regulator of ERα expression and promotes prostate stromal cell activation.	No natural ligand-based activation was examined. Other downstream pathways were not studied. The fibroblast sample size was limited.
Santolla et al., 2015 (87)	Breast cancer associated fibroblasts (CAFs)	Human	E2, G1	E2 (100 nM) / G1 (1 μM), 2-24 h; AG1478 (EGFR inhibitor: 10 μM); PD98059 (MEK inhibitor: 10 μM); Sirtinol (SIRT1 inhibitor: 25 μM)	E2/G1 → GPER → EGFR/ERK/c-fos/AP-1 → ↑ SIRT1 → pro-survival effects	Estrogenic GPER signaling, via SIRT1, promotes breast cancer progression and resistance to therapy.	The study used short-term *in vitro* models that may not reflect chronic signaling in the tumor microenvironment and did not provide direct evidence for paracrine effects of GPER–SIRT1–activated fibroblasts on tumor cells.
Vivacqua et al., 2015 (88)	Breast cancer associated fibroblasts (CAFs)	Human	E2, G1	E2 (100 nM); G1 (100 nM); PD98059 (1 µM); Wortmannin (1 µM), 3 h or 2 h	E2/G1 → GPER → PI3K & ERK1/2 → ↑ Elk1→ ↑ miR144 → ↓ Runx1 → cancer stimulation	Estrogenic GPER-mediated cancer stimulation in cancer associated fibroblasts.	CAFs are heterogeneous across patients and may respond differently to GPER signaling, and the CAF sample size was limited.
Vivacqua et al., 2018 (89)	Breast cancer associated fibroblasts (CAFs)	Human	E2, G1	E2 (100 nM); G1 (100 nM), 4 h/ 8-24 h	E2 / G1 → GPER → ↓ miR-338-3p → ↑ c-fos → ↑ Cyclin D1 → CAF proliferation and tumor promotion	E2 regulates miR-338-3p toward breast cancer progression.	The CAF sample size was small and not subclassified, despite known CAF heterogeneity, and the upstream molecular steps linking GPER activation to miR-338-3p suppression were not fully defined.
He et al., 2024 (90)	Triple negative breast cancer associated fibroblasts (TNBC)	Human	E2, G15	CAFs cultured with conditioned medium from BT549 or MDA-MB-231 ± E2 (100 nM, 12 h), G15 (100 nM, 12 h), MDL-12330 (adenylate cyclase inhibitor: 20 μM, 12 h), H-89 (PKA antagonist: 30 μM, 24 h). BT549 and MDA-MB-231 cells treated with E2 (100 nM, 12 h), G15 (100 nM, 12 h), or subjected to gene knockdown and co-cultured with CAFs	E2 → GPER → activated cAMP/PKA/ CREB → elevated glutamine synthetase (GLUL) and lactate dehydrogenase B (LDHB) → increased glutamine → cancer cell proliferation, survival, invasion, and chemoresistance	Estrogen activated microenvironmental GPER signaling impacts tumor progression in TNBC.	This study focuses on GPER⁺ CAFs (> 60%) and does not address the roles of other CAF subtypes, such as matrix or inflammatory CAFs. Thus, the heterogeneity of GPER^+^ CAFs remain unexplored.
Liu et al., 2021 (91)	Immortalized breast cancer associated fibroblasts (CAFs)	Human	G1, Tamoxifen (TAM)	TAM (10 nM) and G1 (1 μM) for 15/30 min ± pretreatment with G15 (1 μM). Cells were cultured with conditioned medium supplemented with G1 (1 μM) or TAM (10 nM) ± G15 (1 μM) or LY294002 (10 μM) for 24 h. See references for more detail	CAF (G1/TAM → GPER → PI3K/AKT → increases HMGB1) → breast cancer cells (MEK/ERK → increases autophagy → TAM resistance)	GPER as a target to treat ERα- positive breast cancers.	Immortalized lines may not fully reflect the heterogeneity of CAFs found in different breast cancer subtypes. CAF-secreted HMGB1 acts on MCF-7 cells via conditioned media, but did not directly track HMGB1 transfer or uptake by tumor cells.
Yuan et al., 2015 (92)	Breast cancer associated fibroblasts (CAFs)	Human	G1, Tamoxifen (TAM)	TAM: 1 μM, 24 h; G1: 10 nM, 24 h; G15: 1 μM, 24 h; Inhibitors: AG1478 (EGFR: 6 μM); U0126 (MAPK/ERK: 10 μM) or Wortmannin (PI3K: 10 μM)	CAF (fibronectin) → α5β1/β1-integrin (tumor cell) → FAK/Src → PI3K/AKT → EMT/migration, while TAM via GPER→EGFR→ERK boosts β1-integrin expression, potentiating the CAF effect	CAF-tumor cell crosstalk promotes tumor progression and tamoxifen resistance.	CAF signaling upstream of fibronectin was not dissected out.
Yu et al., 2017 (93)	Breast cancer associated fibroblasts (CAFs)	Human	E2, G1, Tamoxifen (TAM)	E2 (100 nM); G1 (100 nM) and TAM (100 nM/ 10 μM), 30 min and 3, 12 and 24 h. For detailed treatment, see references	Tumor cell conditioned medium induced PI3K/AKT → CRM1 mediated nuclear export of GPER in CAFs → cytoplasmic GPER → cAMP/PKA/CREB → pyruvate dehydrogenase kinase 4 (PDK4) and lactate dehydrogenase B (LDHB) expression → pyruvate and lactate production → taken up by tumor cell conferring drug resistance	Cytoplasmic GPER in CAFs provides metabolic support to tumor cells making them drug resistant. Disruption of this metabolic coupling would help in the prognosis and treatment of breast cancer.	The PET/CT findings demonstrate a correlation between high GPER expression in fibroblasts and increased tumor glucose uptake; however, they do not establish a cause-and-effect relationship between fibroblast GPER activity and tumor metabolic behavior.

This table summarizes studies examining GPER signaling in fibroblasts across different tissues and species. The table details ligands or modulators, experimental conditions, downstream signaling pathways, functional outcomes, and study-specific limitations.

**Table 3:** Characteristics of dual ER and GPER pathways in fibroblasts.

Author/ Year	Fibroblast origin	Species	Ligand/ Modulator	Dose/concentration/ treatment exposure	Signaling Pathway/outcome	Relevance	Limitations
Ozawa et al., 2021 (94)	Vocal fold fibroblasts	Male Sprague–Dawley rats	E2	E2: 10^-7^, 10^-8^, or 10^-9^ M, 4–24 h; TGFβ1: 10 ng/mL; ICI 182,780 (ERα antagonist: 10^⁻7^ M); G36 (GPER antagonist: 10^⁻7^ M)	E2 (10^−8^ M) + TGFβ1 → ERα and GPER → ↑ Smad7 and ↓ Col1a1	Anti-fibrotic effects of E2 by modulating TGFβ1/Smad signaling via ERs in vocal fold fibroblasts.	Though E2 decreased collagen I expression, it did not suppress myofibroblast differentiation and the study relied on male rat fibroblasts in short-term *in vitro* culture.
Wang et al., 2020 (95)	Uterosacral ligament fibroblasts	Human	E2	E2: 0, 10^-6^, 10^-7^, 10^-8^, and 10^-9^ mol/L; 0, 24, 48, 72 and 96 h	E2 → ERα/ERβ/GPER activation → ↓ Mfn2 → ↑ cyclin D1 → ↑ fibroblast proliferation and procollagen synthesis	E2 improves pelvic organ prolapse symptoms.	Small sample size and Mfn2–ER receptor interactions not fully elucidated.
Pomari et al., 2015 (96)	Dermal fibroblasts	Human	E2	E2: 1- 100 nM, 4-48 h or 12 days	E2 → ERα / ERβ / GPER1 activation → ↑ fibroblast migration + contraction (no α-SMA induction) → enhanced wound healing via intracrine / paracrine estrogen signaling	E2 directly modulates fibroblast behavior critical for skin repair.	ER signaling downstream pathway is not well characterized.

This table summarizes studies in which estrogenic ligands activated both classical ERs (ERα/ERβ) and GPER in fibroblasts. The table includes fibroblast origin, species, ligands, experimental conditions, signaling pathways, biological outcomes, and experimental limitations.

**Table 4:** Characteristics of phytoestrogen-driven signaling in fibroblasts.

Author/ Year	**Fibroblast origin**	**Species**	**Ligand/ Modulator**	**Dose/concentration/ treatment exposure**	**Signaling Pathway/outcome**	**Relevance**	**Limitations**
Lieben Louis et al., 2019 (97)	Cardiac fibroblasts	Sprague–Dawley rats	Resveratrol	Resveratrol: 5–60 µM, 24 h; pre-treatment with 1 μM E2, or 250 μM Tamoxifen or 100 nM Fulvestrant, 30 min	Resveratrol → ERα independent pathway → inhibited fibroblast proliferation and induced apoptosis	Cardioprotective role of reseveratrol by selective cytotoxicity towards cardiac fibroblasts sparing cardiomyocytes.	High concentrations of resveratrol, estradiol and tamoxifen was used. While ER–independent action was confirmed, other potential mediators were not investigated.
Savoia et al., 2018 (98)	Dermal fibroblasts	Human	E2, Genistein	H_2_O_2_ pre-treatment: 200 μM, 30 min ± pre-stimulation with inhibitors (Fulvestrant (ER), G15 (GPER), L-NAME (NOS), Wortmannin (PI3K), SB203580 (p38MAPK), UO126 (ERK1/2): equimolar, 15 min) ± Genistein: 100 μM, 1 µM and 10 nM (30 min/24 h/70 h)/ E2: 100 nM, 10 nM and 100 pM (30 min/24 h/70 h)	E2/genistein → ER/GPER → p38MAPK, Akt, and ERK1/2 → ↓ iNOS/eNOS activation (→ ↓ NO release); ↓ ROS production; ↑ GSH levels; ↓ MMP-1/9; ↑ mitochondrial membrane potential	Protective effects of E2/genistein under peroxidative stress.	ER subtypes involved were not dissected, and the high genistein dose used induced cytotoxic effects that are unlikely to occur at physiological concentrations.
Zhao et al., 2015 (99)	Newborn dermal fibroblasts	Human	Daidzein	Daidzein: 0.5 μg/mL, 5 μg/mL and 50 μg/mL, 24 or 48 h	Daidzein → ↑ TGFβ → ↑ p-Smad2 / p-Smad3 → ↑ COL1A2 transcription → ↑ collagen synthesis → ↓ MMP1/MMP2 → collagen accumulation	Daidzein exerts a protective effect against skin aging by promoting collagen synthesis and inhibiting collagen degradation.	Fibroblasts were derived from newborn skin, limiting applicability to aged or adult conditions.
Nanashima et al., 2018 (100)	Female dermal fibroblast cell line (TIG113)	Human	Blackcurrant extract (BCE) and four anthocyanins: Cyanidin-3-glucoside (C3G), Cyanidin-3-rutinoside (C3R), Delphinidin-3-glucoside (D3G), and Delphinidin-3-rutinoside (D3R)	BCE: 1.0 or 10.0 µg/mL, 24 h or 48 h. Individual anthocyanins: 10 µM, 24–48 h. E2: 1 nM, 24–48 h	BCE/ anthocyanins → ERα → ↑ COL1A1, COL3A1, ELN, HAS3, TIMP3 mRNA and protein and ↓ MMP12, HYAL3 mRNA → ↑ Collagen type I and III, Elastin and Hyaluronic acid synthesis → enhanced extracellular matrix integrity	Blackcurrant anthocyanins as novel phytoestrogens in human skin fibroblasts.	The fibroblast model (TIG113) lacked ERβ expression; therefore, only ERα-mediated estrogen signaling was evaluated, limiting insight into ERβ- or GPER-related pathways. Since BCE is a mixture of multiple anthocyanins and polyphenols, specific active compounds and their relative contributions remain unclear.
Shin et al., 2017 (101)	Dermal fibroblasts	Human	Soy extract (SE) + Haematococcus extract (HE) → SHM	SHM pre-treatment: 2.5, 5, or 10 µg/mL for 1 hour before UVB exposure: 0.02 J/cm²	SHM → ↓ MAPK phosphorylation → ↓ AP-1 transactivation → ↓ MMP-1 expression	SHM prevents UVB-induced skin wrinkling.	Precise molecular target of SHM in the MAPK pathway is not determined.
Kaňuchová et al., 2021 (102)	Dermal fibroblasts	Human	Genistein	Genistein: 10, 100 and 1000 nM/mL ± TGFβ1: 30 ng/mL, 9 days	TGFβ1→ canonical SMAD and non-canonical AKT, ERK1/2, ROCK → ↑ α-SMA and ↑ fibronectin. GEN→ ERK1/2 expression. GEN+ TGFβ1 → inhibits ERK1/2 and ROCK signaling	Genistein’s anti-fibrotic and wound-healing potential in normal fibroblasts, showing no inhibition of TGFβ1–induced myofibroblast formation.	Small sample size, and specific receptor involved is undetermined.
Kwon et al., 2024 (103)	Lung fibroblasts from idiopathic pulmonary fibrosis (IPF) patients	Human	Genistein	TGFβ1: 5 ng/mL ± genistein: 20 μM, 1 h/24 h	Genistein → ↓ Smad2/3 phosphorylation, ↓ p38MAPK activation, ↓ Collagen I and α- SMA → ↓ fibroblast activation	Genistein act as an anti-fibrotic agent by inhibiting fibroblast activation.	Mechanistic detail is limited, as no validation using pathway inhibitors or gene knockdown approaches was performed. In addition, ER signaling was not directly characterized.
Lu et al., 2024 (104)	Keloid fibroblasts	Human	Genistein	Genistein: 40 to 200 μM, refer to article for more detailed method.	Genistein → ↑ p53 → cell cycle arrest at G2/M phase, ↓ Cdk1/2, ↓ PCNA and Ki67 (proliferation markers) → ↓ CTGF (connective tissue growth factor), ↓ COL1A1 and FN → ↓ migration. Genistein → enhanced endocytosis and ↓ growth factors (FGFR1, VEGFR1, PDGFRα, and PDGFRβ)	Anti-fibrotic and anti-migratory action via selective inhibition of keloid fibroblast by suppressing CTGF axis.	High concentration of genistein and small patient sample size.
Qin et al., 2015 (105)	Cardiac fibroblasts	Rat	Genistein	TGFβ1 (10 ng/mL) ± Genistein (100 μM), 24 h	Genistein → ↑ MTA3 → ↓ TAK1/MKK4/JNK → ↓ proliferation, collagen production and myofibroblast transformation	Genistein mediates its anti-fibrotic effect through an estrogenic, non-Smad signaling axis, providing protection against cardiac diseases related to fibrosis.	Neonatal rat fibroblasts were used; hence, translation to adult human cardiac fibroblasts is uncertain. Isoform-specific ER signaling was not analyzed, and findings were not validated in human cardioblasts.
Leite et al., 2023 (106)	Dermal fibroblasts adult (HDFa) cell line	Human	Biotransformed soybean extract (BE) (identified daidzein and genistein)	BE: 1.33 μg/mL	BE → ERβ → ↑ collagen I	Introduction of BE as a nutricosmetic targeting ERβ to mitigate hypoestrogenism- related skin aging.	Mechanistic detail downstream of ERβ activation is limited, and since the extract contained multiple components, the increase in collagen expression cannot be solely attributed to isoflavones such as genistein.

This table summarizes experimental studies investigating phytoestrogen-mediated signaling in fibroblasts, including fibroblast origin and species, ligand, experimental conditions, downstream signaling pathways, functional effects, and methodological limitations.

**Table 5:** Characteristics of SERM-mediated signaling in fibroblasts.

Author/ Year	Fibroblast origin	Species	Ligand/ Modulator	Dose/concentration/ treatment exposure	Signaling Pathway/outcome	Relevance	Limitations
Aguado et al., 2020 (107)	Dermal fibroblasts from recessive dystrophic epidermolysis bullosa (RDEB) patients	Human	Raloxifene	Raloxifene: 0.2 nM/ 5 μM, 48 h. N-acetylcysteine: 100 μM/ 1 mM, 48 h. Losartan (control drug): 10 μM, 48 h. TGFβ1 stimulation: 1 ng/mL for 3 h to stimulate ALK1/Smad1/5 pathway or 10 ng/mL for 24 h to stimulate ALK5/Smad2/3 pathway	Raloxifene → increases endoglin → downregulates TGFβ1/ ALK/ SMAD signaling → decreases fibrosis and inflammation	Anti-fibrotic effect of raloxifene makes it as a therapeutical candidate for treating RDEB.	Limited patient sample size and mechanistic understanding.
Kim et al., 2022 (108)	Induced pluripotent stem cell (iPSC)-derived fibroblasts and fibroblasts from systemic sclerosis patients	Human	Raloxifene, Bazedoxifene	Raloxifene: 5 µM, 10 µM, 20 µM, 24-48 h ± TGFβ1; Bazedoxifene: 10 µM – 20 µM, 24-48 h ± TGFβ1	TGFβ1 → SMAD2/3 phosphorylation → ↑ α-SMA + collagen I/III → fibroblast-to-myofibroblast differentiation. Raloxifene (and Bazedoxifene) ↓ p-SMAD2/SMAD2 ratio and ↓ GSK-3α/β phosphorylation → ↓ fibroblast proliferation, ↓ collagen content ↓ α-SMA expression, ↓ skin-equivalent thickness	Raloxifene exerts anti-fibrotic effects in systemic sclerosis by targeting TGFβ1/SMAD and GSK-3β signaling.	Small sample size and ER involvement is not dissected.
Wang et al., 2018 (83)	Fibroblast cell line	Not mentioned	Tamoxifen (TAM)	TAM: 10 µM, 0, 12, 24, and 48 h	Tamoxifen → ↓ p-AKT → ↓ Cyclin D1 + ↑ P21/P27 → G1 arrest → ↓ fibroblast proliferation	Tamoxifen shows anti-fibrotic potential by reducing epidural fibrosis.	Mechanistic confirmation of AKT involvement is lacking; the fibroblast origin was not specified, limiting reproducibility, and findings require validation in human fibroblasts.
Turczyk et al., 2017 (109)	Breast cancer associated fibroblasts (CAFs)	Human	4-hydroxytamoxifen (OHT)	Fibroblast growth factor 7 (FGF7: 10 ng/ml) and/or OHT (1 μM)	FGF7/FGFR2-signaling activates PI3K/AKT pathway → ER phosphorylation at Ser167 → ER ubiquitination, proteasomal degradation and ↑ Bcl-2 → FGFR2-dependent resistance to tamoxifen	FGF7/FGFR2-signaling reverse tamoxifen-driven ER stabilization and results in resistance of breast cancer cells to endocrine therapy.	The patient cohort was heterogeneous in treatment regimens, which prevented analysis of FGFR2’s impact on tamoxifen response or overall survival.
Maggiolini et al., 2015 (110)	Breast cancer associated fibroblasts (CAFs)	Human	E2, G1, PBX1, PBX2	100 nM E2 or 1 μM G1 ± PBX1/ PBX2 (10 μM), 10 min	PBX1/ PBX2 → GPER → ↓ phosphorylation of EGFR/ ERK1/2 → ↓ c-fos and CTGF expression → abolished migration of CAFs	Leads for the development of specific GPER antagonists and helps in targeting GPER-dependent tumors.	Limited mechanistic detail or did not explore other downstream pathways of GPER. No cytotoxicity test were done on fibroblasts. Small patient sample size.

This table summarizes experimental studies investigating SERM-mediated signaling in fibroblasts, including fibroblast origin and species, ligand, experimental conditions, downstream signaling pathways, functional effects, and methodological limitations.

Most CAF studies utilized tumor-associated interaction models, including co-culture or paracrine approaches, to examine fibroblast–tumor cell signaling. One study utilized an organotypic mammary duct model comprising a collagen-embedded duct structure lined with MCF7 breast cancer cells, surrounded by matrix with or without primary human mammary fibroblasts (62). Among the studies investigating SERMs, two novel compounds were identified (110). Additionally, in three studies examining phytoestrogens, plant extracts were employed in place of isolated, structurally characterized phytoestrogen compounds (100, 101, 106).

#### 3.1.1 Classical ER-dependent signaling in fibroblasts

Thirty-two studies examined E2-mediated signaling in fibroblasts across various tissues and species (**Table 1**). Of these, one study used ERB041 (ERβ agonist) (47), and two studies used 2-methoxyestradiol (2ME2), a natural E2 metabolite (65, 66), as ligands. E2 exerts anti-fibrotic effects by inhibiting the TGF-β/small mother against decapentaplegic (SMAD) pathway. In dermal fibroblasts from systemic sclerosis patients, E2 suppressed TGFβ-induced collagen synthesis and myofibroblast differentiation through ERα- and ERβ-dependent, proteasome-mediated degradation of phosphorylated SMAD2/3 (p-SMAD2/3), thereby attenuating canonical SMAD signaling (46). These findings have been extrapolated to explain the greater fibrosis severity observed in men and postmenopausal women with reduced E2 levels, although fibroblasts from these populations were not directly studied. In intestinal fibroblasts, ERβ activation inhibited both TGFβ/SMAD signaling and toll-like receptor 4 (TLR4)/myeloid differentiation primary response 88 (MyD88)/nuclear factor kappa B (NF-κB) signaling, exerting anti-fibrotic effects. This was demonstrated only in the “CCD-18Co” cell line (47), which may not fully recapitulate *in vivo* fibroblast behavior. In cardiac fibroblasts, membrane-associated ERβ inhibited angiotensin II (AngII)-induced phosphorylation and nuclear translocation of SMAD3 (48), although a direct mechanistic link between ERβ and SMAD was not established. Similarly, E2 blocked AngII- or endothelin-1 (ET-1)–induced ERβ-dependent activation of cyclic adenosine monophosphate (cAMP)/protein kinase A (PKA) and 5’ AMP-activated protein kinase (AMPK), leading to Ras homolog family member A (RhoA) inactivation and reduced Rho kinase activity, downregulation of TGFβ and connective tissue growth factor (CTGF) in neonatal rat fibroblasts (49). In subsynovial fibroblasts from postmenopausal women, E2 activated ERα and inhibited TGFβ-responsive gene transcription, reducing collagen I/III expression, though the study employed a supraphysiological E2 dose and lacked male or premenopausal controls (50). In three additional studies, specific ERs were not identified; nonetheless, E2 inhibited TGFβ1 signaling through decreased SMAD2 phosphorylation and RhoA- Rho associated coiled-coil containing protein kinase 2 (ROCK2) expression in rat tunica albuginea-derived fibroblasts (51), suppressed signaling pathways mediated by SMAD and mitogen-activated protein kinase (MAPK), including p38 and extracellular signal-regulated kinases (ERK), in human tenon fibroblasts (52), and upregulated cell division cycle 42 (Cdc-42)-dependent anti-fibrotic pathway in immortalized mouse cardiac fibroblasts (53). Further, the effect of E2 under oxidative stress was studied. In human dermal fibroblasts, E2 reduced reactive oxygen species (ROS) via increasing antioxidant response element (ARE)/nuclear erythroid 2-related factor 2 (NRF2) transcription system under oxidative stress induced by both H_2_O_2_ and rotenone (mitochondrial) (54, 55). However, the mechanistic link between NRF2 and ROS, and specific ER subtype remained incompletely defined.

Pro-fibrotic effects of E2 through ERα were identified in two studies. Baker frost et al., demonstrated that E2 induced TGFβ1 and TGFβ2 upregulation by MAPK activation in healthy dermal fibroblasts (56). While in dermal fibroblasts from systemic sclerosis patients, Baker frost et al., identified a positive feedback loop between interleukin (IL)-6 and E2 (57); however, downstream ERα signaling was not explored. Sex specific regulation of collagen expression via E2 and ER signaling was revealed in cardiac fibroblasts, where ERα acted as a repressive factor and ERβ as an inducing factor in females and males, respectively. In females, E2 mediated the serine 118 phosphorylation of ERα that resulted in ERα/ERβ heterodimer formation, which translocated to the nucleus and bound to ERE within the collagen promoter. In males, serine 105 phosphorylation of ERβ resulted in homodimer formation upon E2 stimulation, followed by nuclear translocation and binding to the promoter sequence. Although this regulation was conserved across human and rat, the mechanistic insights were derived solely from rat fibroblasts (58).

The tumor microenvironment can influence tumor progression through estrogen signaling in CAFs. E2 stimulated CAFs to secrete stromal-derived factor-1 (SDF-1α), which recruited myeloid-derived suppressor cells (MDSCs) to the tumor (59); however, this study used a supraphysiological E2 dose and did not explore the ER involved. In another study, E2 acted through ERα-mediated upregulation of extracellular matrix metalloproteinase inhibitor (CD147 or EMMPRIN) in CAFs co-cultured with cancer cells, thereby promoting cancer migration and invasion (60), although only a limited number of CAF lines were tested and downstream signaling remained incompletely mapped. E2-induced secretion of midkine (MDK) in ER+ ovarian fibroblasts facilitated gastric cancer metastasis via the MDK-low-density lipoprotein receptor-related protein 1 (LRP1) axis (61), yet the small sample size and lack of mechanistic insights into how estrogen triggers MDK secretion limit interpretation. Using an organotypic human mammary duct model, Morgan et al. demonstrated that fibroblasts modulated ER signaling by enhancing ER transactivation and reducing ER protein levels in MCF7 cells grown in co-culture, which decreased apoptosis and induced hyperplasia (62). Although E2 acted via ER to reduce apoptosis in cancer cells, in fibroblasts, it appeared to signal through a non-ER mechanism that needs to be elucidated. Also, the use of high E2 doses and a low-throughput model design further constrained the study.

E2 exhibits protective effects on human uterosacral ligament fibroblasts and improves pelvic organ prolapse (POP). E2 ameliorated mechanical stress-induced cell apoptosis and cell death through ERα-mediated upregulation of poly-ADP-ribose polymerase (PARP1) and B-cell lymphoma 2 (Bcl-2) (63); however, the small sample size, short culture duration, lack of mechanistic validation of PARP1-ERα signaling, and the short half-life of E2 may limit the interpretation of this effect. E2 via ERα-mediated repression of TGFβ1 signaling in nipple fibroblasts, reduced fibroblast-derived inhibition of epidermal proliferation, thereby contributing to specialized nipple epidermis formation (64); however, receptor specificity and downstream signaling were not detailed.

The E2 metabolite 2ME2 exerted anti-proliferative and pro-apoptotic effects in keloid fibroblasts through downregulation of the p38 mitogen-activated protein kinase (p38 MAPK)/cellular Myc (c-Myc)/Activating Transcription Factor-2 (ATF2)/Jun proto-oncogene (c-Jun) signaling axis (65) and caspase-dependent reduction in the Bcl-2/Bcl-2–associated X protein (Bax) ratio (66). Both studies, however, did not identify a specific ER involved and were limited by small sample sizes. Three studies identified anti-inflammatory roles of E2. In synovial fibroblasts from rheumatoid arthritis patients, E2 upregulated 1-methyl nicotinamide, which decreased inflammation by downregulating Signal transducer and activator of transcription (STAT), MMP3/9, and MAPK14 (67), though the receptor mechanism remained unexplored. In mouse embryonic fibroblasts, both 17α-estradiol (17α-E2) and 17β-estradiol (17β-E2) activated ERα, which suppressed NFκB-p65, tumor necrosis factor-alpha (Tnf-α), and Il-6 while increasing Il-4 and Il-6 receptor expression (68); however, high E2 doses were used, and adult fibroblasts were not examined. Song et al. showed that in embryonic fibroblasts from Nrf2 knockout mice, E2 activated Nrf2 binding to antioxidant response elements within the ERβ promoter, restoring ERβ expression and inhibiting NFκB signaling (69); however, this was supported only by in silico prediction of Nrf2-ERβ promoter interactions.

Cheng et al. demonstrated that estradiol (E2) exerts an anti-senescence effect in POP–derived fibroblasts via the sirtuin 1 (SIRT-1)/tumor protein 53 (p53)/cyclin-dependent kinase inhibitor 1A (p21) signaling pathway (70). However, this study did not investigate alternative signaling mechanisms or identify the specific ER mediating this effect.

Several studies have further shown that E2 modulates the expression of microRNAs (miRNAs) in fibroblasts. For instance, E2-loaded nanoparticles (E2-NPs) suppressed the proliferation of cardiac fibroblasts through the downregulation of miR-302b, promoting apoptosis and attenuating inflammation in myocardial infarction (71). Nonetheless, fibroblasts were not directly treated with E2-NPs, and neither the downstream signaling pathways nor the receptor mechanisms involved were delineated. In synovial fibroblasts, E2-induced upregulation of miR-101-3p reduced hyaluronan synthase 2 (HAS2) expression, contributing to the pathogenesis of idiopathic condylar resorption (ICR) (72); however, the small sample size and single-pathway focus in a multifactorial disease limit the generalizability of these findings. Moreover, E2, acting through ERs, was reported to suppress miR-7 expression by inhibiting STAT1 expression and activity, thereby enhancing wound healing in lung and dermal fibroblasts from both young and aged donors (73). This rejuvenating effect effectively restored aged fibroblasts to a more youthful phenotype; however, other E2-related or aging-associated signaling pathways were not examined.

E2 has also been implicated in the modulation of anti-viral responses. In human lung fibroblasts, E2 increased tissue inhibitor of metalloproteinases-1 (TIMP-1) expression via ERα, suggesting that TIMP-1 could serve as a potential biomarker of acute lung injury following severe acute respiratory syndrome coronavirus 2 (SARS-CoV-2) or influenza A infection (74); however, the mechanistic link between ERα activation and TIMP-1 upregulation remains incomplete. In endometrial fibroblasts, E2 initiates antiviral interferon-mediated responses through SDF-1α, thereby preventing human immunodeficiency virus (HIV) infection of CD4^+^ T cells (75). Uterine fibroblasts both produce and respond to IL-27 upon a viral challenge. E2 enhances IL-27 secretion, which further augments response to viral challenge, and also selectively suppresses IL-27-induced indoleamine 2,3-dioxygenase (IDO) expression via ERα while maintaining the induction of antiviral interferon-stimulated genes (ISGs) such as apolipoprotein B mRNA editing enzyme, catalytic polypeptide-like 3G (APOBEC3G) (111). Nevertheless, the downstream ERα–IL-27 signaling axis was not characterized, and the use of a single E2 concentration with short-term exposure does not reflect physiological hormonal fluctuations. In contrast, E2 did not alter IFNλ1 expression or ISG secretion in uterine fibroblasts following viral mimic exposure (76), although the mechanism underlying this E2 insensitivity remains undefined and was examined in a limited sample size.

#### 3.1.2 Rapid non-genomic GPER signaling in fibroblasts

Seventeen studies reported E2-, G1-, or G15-mediated signaling through GPER in fibroblasts derived from various sources, including dermal, cardiac, synovial, and cancer-associated fibroblasts across species (**Table 2**). In cardiac fibroblasts, G1 exerted a protective effect through GPER-activated phosphoinositide 3-kinase/protein kinase B (PI3K/Akt) signaling, reducing apoptosis via downregulation of BAX and Caspase-3 and upregulation of Bcl-2 (77). However, the fibroblast strain was not specified, and the upstream or downstream intermediates of PI3K/Akt remained unexplored. In dermal fibroblasts, E2 induced rapid cytoskeleton reorganization via GPER-mediated extracellular signal–regulated kinases 1 and 2 (ERK1/2) activation (78), although signaling downstream of ERK1/2 was not explored. An anti-fibrotic effect of G1 was shown by inhibiting ERK1/2 and MMP9 in cardiomyocytes, which then reduced TGFβ expression in co-cultured neonatal rat cardiac fibroblasts (79). Notably, fibroblast activation was examined indirectly using cardiomyocyte-conditioned media, and GPER knockout or transgenic models were not employed to confirm mechanistic specificity. In synovial fibroblasts from frozen shoulder patients, E2 and G1 activated GPER-dependent PI3K/Akt signaling, leading to reduced fibroblast activation and collagen deposition (80). Nevertheless, the study’s small sample size and unreported concentration of MK-2206 (Akt inhibitor) limit generalizability. Further evidence indicates that GPER functions as an anti-fibrotic mediator by inhibiting TGF-β1/SMAD2/3 phosphorylation and inducing SMAD7 expression in cardiac fibroblasts (81). However, the *in vitro* concentration of G1 was not specified, and the role of GPER was not validated using antagonist or knockdown approaches. GPER also reduced fibrosis by downregulating the cyclin-dependent kinase 1 (CDK1)/cyclin B1/α smooth muscle actin (α-SMA)/TIMP pathway in cardiac fibroblasts (82), yet only male-derived fibroblasts were examined, and the downstream pathways linking GPER activation to CDK1/cyclin B1 suppression remain incompletely defined. Finally, G1 inhibited inducible nitric oxide synthase (iNOS) expression and nitric oxide (NO) synthesis via GPER activation, further supporting an anti-fibrotic role of this receptor in cardiac fibroblasts (83).

Our research identified several studies indicating that CAFs promote cancer progression primarily through GPER-mediated signaling. In breast cancer, E2/G1-driven engagement of GPER established a positive regulatory loop in which IL1β produced by CAFs increased the expression of its cognate receptor, IL1R1, in cancer cells. This interaction led to upregulation of IL1β/IL1R1 target genes such as prostaglandin E synthase-1 (PTGES), cyclooxygenase-2 (COX2), receptor for advanced glycation end products (RAGE), and ATP-binding cassette G2 (ABCG2), thereby promoting a pro-tumorigenic inflammatory phenotype (84). However, the findings were constrained by a small sample size. In another study involving CAF from breast cancer, Luo et al. showed GPER mediated both rapid signaling, characterized by increased intracellular Ca²^+^ and ERK1/2 phosphorylation, and slower responses including adhesion, spreading, proliferation and migration (85). Nonetheless, the underlying mechanistic details were not fully elucidated. GPER activation promoted stromal cell activation via suppressing ERα and its downstream signaling in CAF from prostate cancer (86); however, this study employed a small sample size and relied solely on GPER overexpression or small interfering RNA (siRNA) knockdown without using a natural ligand. E2 and G1 exerted pro-survival effects by the induction of SIRT1 expression through GPER and the subsequent activation of the epidermal growth factor receptor (EGFR)/ERK/cellular fos (c-fos)/activator protein 1 (AP-1) transduction pathway (87); however, the study was a short-term culture, and evidence for paracrine effects of GPER-SIRT1-activated fibroblast on tumor cells was not determined. Two studies further implicated miRNAs in GPER-mediated cancer progression. Through the PI3K/ERK1/2/ETS-like transcription factor 1 (Elk1) transduction pathway, GPER induced miR144 expression, leading to Runt-related transcription factor 1 (Runx1) downregulation in CAF from breast cancer (88), though the small cohort size and CAF heterogeneity limited interpretability. In another study in CAFs from breast cancer, GPER activation downregulated miR-338-3p, resulting in increased c-Fos and cyclin D1 expression (89); however, the upstream link between GPER and miR-338-3p was not fully defined. Moreover, estrogen-activated GPER in CAFs upregulated glutamine synthetase (GLUL) and lactate dehydrogenase B (LDHB) expression via the cAMP/protein kinase A (PKA)/cAMP response element-binding protein (CREB) signaling, facilitating glutamine production that mediates metabolic coupling between CAF and breast cancer cells (90). Nevertheless, the heterogeneity of GPER+ CAFs remained unexplored.

GPER-mediated mechanisms in CAFs also contribute to chemoresistance in cancer cells. In these studies, CAFs from breast cancers were stimulated with G1 or Tamoxifen (TAM), a SERM. Liu et al. showed that TAM resistance was mediated through the GPER/PI3K/AKT signaling pathway in CAFs, promoting high mobility group box 1 (HMGB1) expression and secretion (91). Yuan et al. indicated a GPER/EGFR/ERK signaling that upregulated β1-integrin expression and activated downstream kinases, contributing to CAF-induced cell migration and epithelial-mesenchymal transition, in MCF-7R cells (92). Yu et al. further demonstrated that tumor cell–activated PI3K/AKT signaling induced Chromosome Region Maintenance 1 (CRM1)-dependent cytoplasmic translocation of GPER in CAFs, activating an estrogen/GPER/cAMP/PKA/CREB signaling cascade driving pyruvate and lactate production taken up by tumor cells (93). However, Liu et al. used immortalized lines that may not fully reflect the CAF heterogeneity, and although they showed that CAF-secreted HMGB1 acted on MCF-7 cells via conditioned media, they did not directly track HMGB1 transfer or uptake by tumor cells (91). Further, Yuan et al. did not completely decipher the signaling mechanisms within CAFs (92), and Yu et al. failed to establish a cause-and-effect relationship between fibroblast GPER activity and tumor metabolic behaviour (93).

#### 3.1.3 Dual ER and GPER pathways in fibroblasts

Three included studies activated both ER and GPER pathways (**Table 3**). E2 modulated TGFβ1/SMAD signaling by activating both ERα and GPER to decrease collagen I expression in male vocal fold fibroblasts (94); however, myofibroblast differentiation was not suppressed. Another study on uterosacral ligament fibroblast reported a protective effect of E2 through activation of ERα/ERβ/GPER, which reduced Mitofusin-2 (Mfn2) expression (95); however, the Mfn2-ER interactions were not fully deciphered, and the study also involved a small sample size. In another study, Pomari et al. demonstrated that E2 enhances wound healing in dermal fibroblasts by promoting fibroblast migration and contraction without inducing α-SMA expression, an effect mediated through ERα, ERβ, and GPER activation (96), although the downstream signaling cascades were not explored.

#### 3.1.4 Phytoestrogen-driven signaling in fibroblasts

We identified ten studies reporting phytoestrogen-mediated signaling in fibroblasts derived from cardiac, dermal, lung and keloid tissues across species (**Table 4**). Although phytoestrogens are known to act via ER-dependent mechanisms (31), resveratrol inhibited fibroblast proliferation and induced apoptosis in rat cardiac fibroblasts through an ERα-independent pathway (97). This outcome may have resulted from the use of relatively high doses of resveratrol, E2 or tamoxifen. Phytoestrogens also appear to exert a protective role under oxidative stress. Genistein attenuated ROS production by modulating endothelial Nitric Oxide Synthase (eNOS)/iNOS-dependent NO and ROS release, glutathione (GSH) content and mitochondrial function through ER- and GPER-mediated PI3K-Akt, p38 MAPK, and ERK1/2 pathways under peroxidative stress (98). Yet, the specific ER subtypes involved were not dissected, and the genistein concentration used exceeded ranges typically applied to model physiologically relevant exposure. Diadzein exhibited a protective effect by promoting collagen synthesis and inhibiting collagen degradation via TGFβ/SMAD signaling in newborn human skin fibroblasts (99); however, extrapolation to aged or adult fibroblasts remains uncertain. Blackcurrant extracts and anthocyanins demonstrated phytoestrogenic activity in human skin fibroblasts through ERα activation, increasing collagen I/III, elastin and hyaluronic acid synthesis, thereby improving extracellular matrix integrity (100). Because the fibroblast model (TIG113) used lacked ERβ expression, only ERα-mediated signaling was evaluated, limiting insight into ERβ- or GPER-related pathways. Moreover, the specific active compounds within the extract and their relative contributions were not identified. In another study, phytoestrogen-rich extracts from soybean and Haematococcus protected against UVB-induced dermal, reducing MMP1 expression via downregulation of MAPK phosphorylation and AP-1 transactivation (101). The precise molecular target of the extract, however, was not determined.

The anti-fibrotic role of genistein has been examined in several fibroblast models. When combined with TGFβ, genistein inhibited ERK1/2 and ROCK signaling; however, it alone did not prevent TGFβ1-driven differentiation of dermal fibroblast into myofibroblasts (102). This suggests an anti-fibrotic effect of genistein while preserving the normal wound healing process. Nonetheless, the limited number of samples represents a major constraint, and the specific receptor involved remains unidentified. Genistein prevented fibroblast activation in lung fibroblasts by modulating the TGFβ/SMAD pathway through the inhibition of SMAD2/3 phosphorylation and p38MAPK activation (103), but it lacked mechanistic detail, and the receptors involved were not characterized. In keloid fibroblasts, genistein exhibited anti-fibrotic and anti-migratory actions by suppressing TGFβ-induced CTGF, Collagen Type I Alpha 1 chain (COL1A1) and fibronectin expression, while enhancing endocytosis and repressing growth factors via the CTGF axis (104). Nonetheless, high genistein concentrations and small sample size limit interpretation. In cardiac fibroblasts, genistein reduced proliferation, collagen synthesis, and myofibroblast transformation through the regulation of metastasis-associated protein 3 (MTA3)/transforming growth factor-β–activated kinase 1 (TAK1)/mitogen-activated protein kinase kinase 4 (MKK4)/c-Jun N-terminal kinase (JNK) pathway (105), though the use of neonatal rat fibroblasts and unidentified receptor involvement restricts extrapolation. Finally, a biotransformed soybean extract stimulated collagen I production through ERβ in human dermal fibroblasts (106). Despite this, the mechanistic detail downstream of ERβ activation was not explored, and since the extract contained multiple components, the increase in collagen expression cannot be solely attributed to isoflavones such as genistein.

#### 3.1.5 SERM-mediated signaling in fibroblasts

Five studies were included under SERM-mediated signaling (**Table 5**). Raloxifene decreased fibrosis and inflammation in human recessive dystrophic epidermolysis bullosa fibroblasts by increasing endoglin and downregulating TGFβ1/Activin Receptor-Like Kinase (ALK)/SMAD signaling (107). However, the study used a limited sample size and mechanical understanding. In fibroblasts from systemic sclerosis patients, Raloxifene and Bazedoxifene exerted anti-fibrotic effects by repressing p-SMAD2/SMAD2 ratio, glycogen synthase kinase (GSK)-3α/β phosphorylation, resulting in decreased fibroblast proliferation, collagen content, α-SMA expression, and skin-equivalent thickness (108). Nonetheless, the study employed a small sample size and did not delineate ER involvement. Using a fibroblast cell line, Wang et al., demonstrated that tamoxifen reduced epidural fibrosis by downregulating p-AKT and cyclin D1, thereby promoting G1 cell cycle arrest (112). Yet, mechanistic confirmation of AKT involvement was lacking and the fibroblast origin was not mentioned. Breast cancer cells acquired tamoxifen resistance through the Fibroblast Growth Factor 7 (FGF7)/Fibroblast Growth Factor Receptor 2 (FGFR2) signaling axis that activated PI3K/AKT pathway leading to ER-Ser167 phosphorylation, ER ubiquitination, proteasomal degradation and upregulated Bcl-2 expression (109). However, the study included a heterogeneous patient cohort with variable treatment regimens. Finally, Maggioloini et al. identified two novel SERMs, PBX1 and PBX2, that acted via GPER to phosphorylate EGFR/ERK1/2, resulting in decreased c-fos and CTGF expression and inhibition of CAF migration (110). Despite these promising observations, certain studies were limited by small sample sizes, whereas others provided incomplete mechanistic detail, did not explore alternative GPER downstream pathways, or omitted cytotoxicity testing in fibroblasts.

### 3.2 Summary

**Figure 2:**
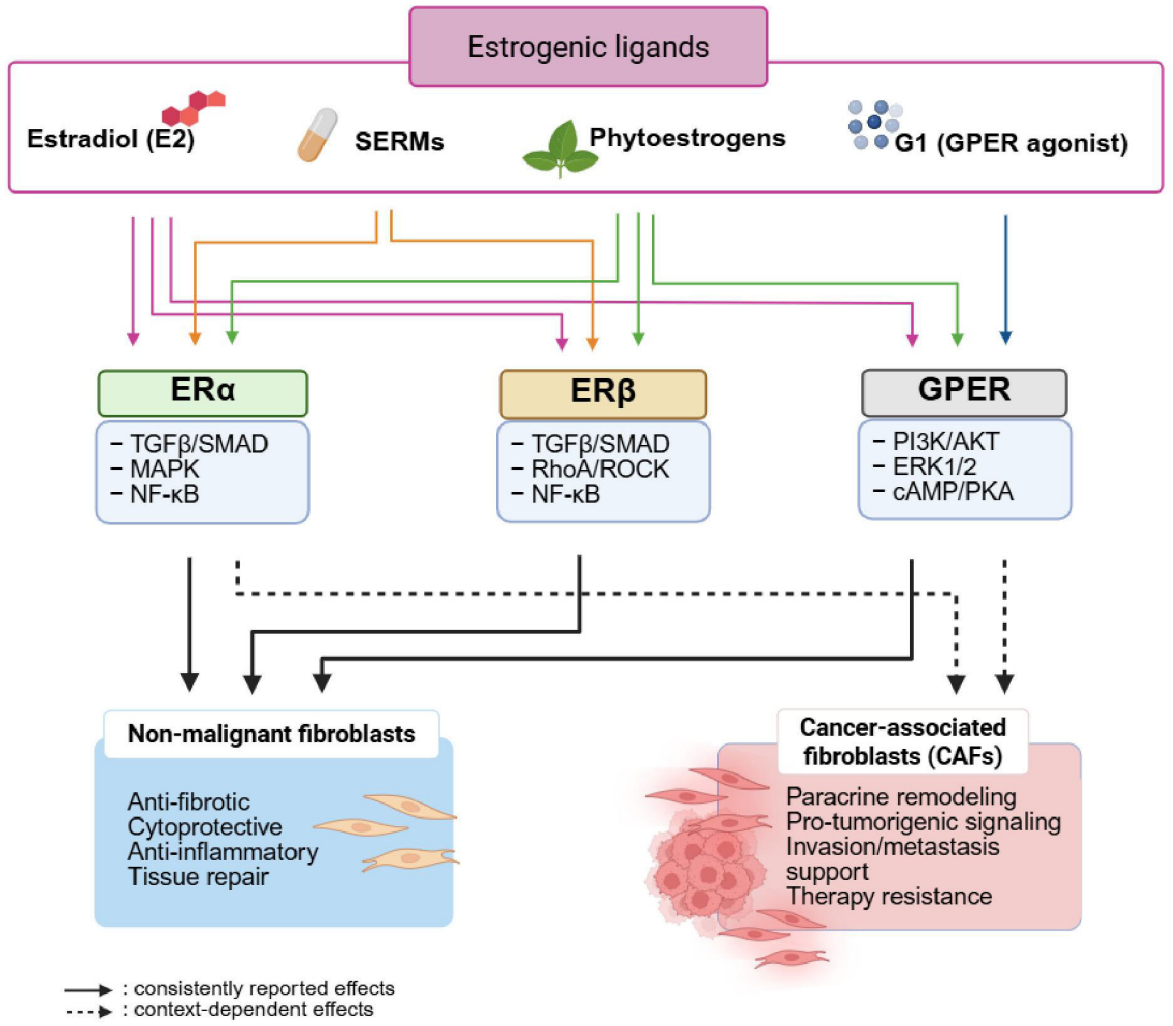
Estrogenic ligands, including estradiol (E2), the selective G protein-coupled estrogen receptor (GPER) agonist G1, selective estrogen receptor modulators (SERMs), and phytoestrogens, engage distinct estrogen receptors (ERα, ERβ, and GPER) to activate downstream signaling pathways in fibroblasts. In fibroblasts from non-malignant tissues (left panel), receptor-mediated signaling is predominantly associated with anti-fibrotic, cytoprotective, anti-inflammatory, and tissue-repair responses. In contrast, in cancer-associated fibroblasts (CAFs; right panel), estrogen signaling is reprogrammed towards paracrine remodeling, pro-tumorigenic signaling, invasion/ metastasis support, and therapy resistance. Ligand-receptor relationships are shown conceptually and do not imply uniform receptor engagement across all contexts. Solid arrows indicated signaling effects consistently reported across multiple studies, whereas dashed arrows denote context-dependent effects.

Overall, this review demonstrates that estrogenic signaling in fibroblasts is diverse and context-dependent. E2, phytoestrogens and SERMs were shown to exert a range of effects, including anti-fibrotic, anti-inflammatory, anti-migratory, pro-fibrotic, wound healing, pro-apoptotic, anti-apoptotic, and pro-survival responses through classical ERs (ERα and ERβ) as well as the membrane-associated receptor GPER. While ERβ- and GPER-mediated pathways were generally associated with protective and anti-fibrotic outcomes, GPER signaling was mainly associated with tumor-promoting characteristics and chemoresistance. Altogether, estrogenic modulation of fibroblasts integrates both genomic and non-genomic mechanisms that can either produce a protective effect or, under pathological conditions, promote fibrosis and tumor progression. However, most studies were limited by small sample sizes, relatively high ligand concentrations, and incomplete receptor-specific validation, underscoring the need for robust mechanistic and translational investigations. A conceptual overview of these receptor-specific signaling patterns and their context-dependent effects is summarized in **Figure 2**.

### 3.3 Measures of quality

The quality of the publications included in this review was evaluated using established bibliometric indicators, including the h5-index, journal impact factor, and SCImago Journal Rank (SJR) quartile classification (**Supplementary Table 2**). Among the 67 included studies, 21 were published in journals with an h5-index greater than 100, 44 in journals with an h5-index below 100, and 2 in journals without a reported h5-index. The journal impact factors ranged from 1.0 to 15.7, indicating a wide variation in citation influence across the selected literature. Based on SCImago classification, 44 articles (65.7%) appeared in Q1 journals, 19 (28.4%) in Q2, 1 (1.5%) in Q3, and 1 (1.5%) in Q4. Additionally, two studies were published in a journal that has since been discontinued in Scopus (as of 2021).

## 4 Discussion

This scoping review mapped the intracellular signaling pathways modulated by estrogens, SERMs, and phytoestrogens in fibroblasts derived from human, rat, and mouse tissues. Collectively, the evidence demonstrates that E2, SERM and phytoestrogens engage multiple intracellular cascades through ERα, ERβ, and membrane-associated GPER, influencing fibroblast proliferation, differentiation, and tissue remodeling across physiological and pathological contexts.

Anti-fibrotic signaling emerged as the most consistent pattern. In diverse fibroblast lineages, E2 suppressed canonical TGFβ/SMAD signaling and its non-canonical extensions (RhoA–ROCK, MAPK, and NF-κB pathways), thereby attenuating collagen synthesis and myofibroblast differentiation (46–49, 52, 53). These effects were largely mediated by ERβ, although ERα-dependent anti-fibrotic mechanisms were also described, such as in subsynovial fibroblasts and stiffness-driven fibrosis models (50, 113). Further, E2 reduced oxidative stress in dermal fibroblasts by increasing the ARE/Nrf2 transcription system (54, 55). Thus, proving the protective effect of E2. In contrast, a smaller number of studies identified pro-fibrotic ERα signaling in dermal fibroblasts, where E2 enhanced TGFβ1/2 via the MAPK/ERK–EGR1 axis and IL-6 expression through a positive feedback loop that amplifies fibroblast activation (56, 57). Sex-specific findings in cardiac fibroblasts further highlight that ERα exerts repressive, and ERβ activating, effects on collagen synthesis, providing a mechanistic basis for sex differences in cardiac fibrosis (58). In the tumor microenvironment, estrogen signaling in CAFs contributes to paracrine remodeling and tumor progression, in contrast to the predominantly anti-fibrotic and protective effects observed in the fibroblasts from non-malignant tissues. This divergence underscores the context-dependence of estrogen signaling. Studies involving CAFs typically used tumor- associated interaction models including co-culture as well as paracrine approaches, to recapitulate the tumor microenvironment. Contrastingly, studies reporting anti-fibrotic estrogen responses in non-malignant tissues were largely conducted in monoculture system, with only limited use of non-tumor paracrine models, such as cardiomyocytes and cardiac fibroblasts cross-talk (79), which preserved anti-fibrotic outcomes. Within tumor-associated contexts, E2 stimulated the secretion of SDF-1α, CD147, and MDK, enhancing immune evasion, epithelial invasion, and metastasis (59–62). These findings align with broader evidence that stromal ER activity shapes the inflammatory and metabolic landscape of estrogen-responsive tumors (114). Beyond fibrosis and cancer, E2 signaling promotes fibroblast survival, differentiation, and tissue maintenance. Through ERα, E2 upregulates PARP1 and Bcl-2, protecting uterosacral ligament fibroblasts from mechanical stress, and represses TGFβ1 in nipple fibroblasts to support epidermal remodeling (63, 64). E2 also exerts anti-senescence effects via the SIRT1/p53/p21 axis (70), modulates E2-responsive microRNAs that regulate ECM and wound healing (71–73), and, through ERα, enhances TIMP-1 and interferon-stimulated genes, linking estrogen to antiviral and immune homeostasis (74, 75, 111). Taken together, these findings reveal E2 as a pleiotropic regulator of fibroblast biology, integrating anti-fibrotic, cytoprotective, and immunomodulatory signaling through receptor- and tissue-specific mechanisms. While the TGFβ/SMAD pathway remains the most frequently implicated target, variability in ER subtype activation, experimental models, and hormonal context underscores the need for standardized approaches. Future studies using primary human fibroblasts, physiological E2 concentrations, and receptor-selective tools will be essential to delineate how estrogenic signaling orchestrates fibroblast behavior across tissues and between sexes.

Evidence from our study revealed that GPER activation mediates both protective and anti-fibrotic effects. In cardiac and skin fibroblasts, GPER acted through PI3K/AKT and ERK1/2 (77, 78). Additionally, GPER mediated a crosstalk between cardiomyocytes and fibroblasts through ERK1/2–MMP-9–TGF-β1 signaling (79). In synovial fibroblasts and cardiac fibroblasts, GPER activation led to a reduction in fibrosis. This occurred via the inhibition of TGFβ1/SMAD, CDK/cyclin/α-SMA/TIMP and iNOS expression (81–83). Across the included studies, which were predominantly conducted in CAFs, selective activation of GPER using G1 generally elicited functional responses comparable to those observed with E2. However, the concentrations required to achieve these effects varied between studies, with some reporting similar responses at different ligand concentrations (80, 84, 85, 87) and others using comparable doses (78, 88, 89, 93). GPER signaling in CAFs has been associated with tumor progression. In CAFs derived from breast and gastric cancer patients, GPER-mediated signaling induced proliferation, adhesion, invasion and migration while rendering chemoresistance to the cancer cells (84–93). Thus, GPER-mediated signaling is exemplified in CAFs, highlighting its role in the tumor microenvironment promoting cancer and therapy resistance. Therefore, targeting GPER may represent a therapeutic approach to treat cancer.

Beyond distinct ER- or GPER-mediated mechanisms, several studies demonstrate that E2 can activate both receptor systems concurrently, producing coordinated protective effects in fibroblasts. Through ERα and GPER, E2 mediated anti-fibrotic effects in vocal fold fibroblasts (94), whereas simultaneous activation of ERα, ERβ and GPER was associated with improved POP (95) and dermal wound healing (96). Collectively, these findings suggest that E2 integrates slow genomic and rapid non-genomic signaling pathways to provide synergistic protection across different fibroblast populations.

Phytoestrogens, owing to their estrogen-like properties (26, 27), protect the fibroblasts against peroxidative stress implicated in skin ageing and fibrosis. E2 and Genistein through ER and GPER and kinases activation protected dermal fibroblasts against peroxidation by regulating oxidant/antioxidant system and mitochondrial membrane potential (98). Similarly, Daidzein exerted anti-ageing effects through TGFβ/SMAD signaling (99), while blackcurrant extract and its anthocyanins promoted collagen synthesis through ERα activation, identifying anthocyanins as a novel phytoestrogen (100). Soy-derived isoflavones have been widely characterized for their estrogenic and protective actions (115). A combination extract of soy and Haematococcus prevented UVB-induced skin wrinkling through inhibition of MAPK phosphorylation and AP-1 transactivation (101), whereas a biotransformed soybean extract, enriched in daidzein and genistein, acted via ERβ to mitigate hypoestrogenism-related skin ageing (106) and is consistent with Seo et al. (115),. Genistein also exhibited anti-fibrotic potential through different signaling cascades across different fibroblasts. In dermal fibroblasts, genistein together with TGFβ1 was able to inhibit ERK1/2 and ROCK signaling (102), in lung fibroblasts, genistein reduced TGF-β1-induced expression of p-SMAD2/3 and p-p38 MAPK (103), in keloid fibroblasts, it suppressed CTGF axis (104) and in cardiac fibroblasts, it regulated MTA3/TAK1/MKK4/JNK signaling pathway (105). Thus, these studies highlight the anti-fibrotic potential of genistein in normal and diseased fibroblasts contexts. Besides these, resveratrol, a phytoestrogen, showed specificity for ERα (115), however, in cardiac fibroblasts, its cardioprotective effects were mediated through an ERα-independent pathway (97). This conflict could be due to high concentrations of resveratrol, E2 and tamoxifen used in this study. In general, the evidence indicates that phytoestrogens exert a protective effect under stress and improve skin conditions by modulating both classical ER-dependent and alternative signaling pathways, supporting their potential as hormone-mimetic therapeutics for estrogen-deficient conditions.

SERMs are compounds with either agonistic or antagonistic activity. Among the studies, we identified only three traditional SERMs, including raloxifene, bazedoxifene and tamoxifen. Raloxifene, bazedoxifene and tamoxifen demonstrated an anti-fibrotic effect through TGFβ1/SMAD and p-AKT/cyclin D1 pathways (107, 108, 112). In cancer contexts, tamoxifen exhibited a growth-inhibitory effect; however, growth factors like FGF7 secreted by CAF counteracted this effect, rendering tamoxifen resistant to cancer cells (109). Thus, CAF secreted growth factors acted in a paracrine manner to render tamoxifen resistance to cancer cells and highlighting the role of the tumor microenvironment. Additionally, two novel SERMs, PBX1 and PBX2, were identified, acting predominantly via GPER (110). This would help in targeting GPER-dependent tumors where traditional ERα/ERβ-directed agents may be less effective. Together, SERMs identified in this study exhibited anti-fibrotic and tumor-modulating properties, underscoring their dual therapeutic relevance in fibrosis attenuation and hormone-responsive cancer management.

## 5 Limitations

Several methodological limitations should be considered regarding this scoping review. A formal assessment of study quality or risk of bias was not conducted as the primary aim of scoping reviews is to map the existing evidence rather than to evaluate methodological rigor. However, the quality of included sources was appraised using bibliometric indicators, such as journal impact factor and SCImago Journal Rank quartiles, to provide contextual information on the credibility and influence of the evidence base (**Supplementary Table 2**). Across the included studies, mechanistic details of ER- and GPER- signaling were often insufficient to describe the complete molecular pathway involved. High concentrations of ligands or modulators were frequently used, restricting extrapolation of results to real-world conditions. In addition, receptor characterization was often incomplete, making it difficult to delineate which ER subtype mediated the observed effects under normal or diseased states. Some studies relied on neonatal rat fibroblasts, which may not accurately reflect adult human fibroblast physiology due to developmental and interspecies differences. Furthermore, small sample sizes and limited patient cohorts reduce the generalizability of findings, while occasional omission of critical experimental details, such as ligand or inhibitor concentration (80, 81) or cell line species (112), impedes reproducibility. Considerable heterogeneity across species, tissue origins and experimental designs also complicates direct comparison between studies and limits mechanistic integration. Most of the included studies investigated fibroblasts from female donors and very few studies were restricted to male-derived fibroblasts. Only one study explicitly compared male and female cardiac fibroblasts and reported an opposing role of ERα and ERβ in collagen gene expression, highlighting sex-specific differences in estrogen signaling (58). These findings underscore an important gap in the systematic investigation of sex-dependent differences in fibroblast estrogen signaling. Collectively, these limitations warrants the need for standardized experimental designs, physiologically relevant models, receptor-specific validation and balanced inclusion of male and female fibroblast models to improve mechanistic clarity and generalizability across tissues and disease contexts.

Additionally, some methodological limitations of our review should be acknowledged. The literature search was limited to selected databases, English language publications, which may have excluded relevant grey or regional studies. The included studies displayed substantial heterogeneity in experimental design, fibroblast source (dermal, cardiac, uterine, synovial, and tumor-associated), and ligand type (E2, SERMs, and phytoestrogens), which limited direct comparison and mechanistic integration. Despite these limitations, this review provides a comprehensive overview of current evidence, mapping the complex network of ER-mediated signaling in fibroblasts and identifying key mechanistic and methodological gaps that warrant further investigation.

## 6 Future directions

Future studies investigating estrogen signaling in fibroblasts should aim to address several gaps identified in the current literature. Greater standardization of experimental designs, including the use of physiologically relevant ligand concentrations and comprehensive receptor-specific characterization, would improve comparability across studies and strengthen mechanistic interpretation. Increased use of primary human fibroblasts, alongside careful consideration of species- and tissue-specific differences, may enhance the translational relevance of experimental findings. Importantly, systematic inclusion of sex as a biological variable, with direct comparison of male- and female-derived fibroblasts, is needed to clarify sex-dependent differences in ER–mediated signaling. Finally, integrating fibroblast signaling within the context of tissue microenvironments, particularly in fibrotic and tumor-associated settings, may provide further insight into the context-dependent actions of estrogenic pathways. Together, these approaches may advance mechanistic understanding of estrogen-mediated fibroblast signaling and inform the development of more targeted and context-specific therapeutic strategies.

## 7 Conclusions

This scoping review mapped the current evidence on ER-mediated signaling in fibroblasts across tissues and experimental contexts. The findings revealed that fibroblasts respond to ligands through ERα, ERβ and GPER-mediated mechanisms, which activate both slow genomic and rapid non-genomic signaling pathways such as TGFβ/SMAD, PI3K/AKT, ERK1/2, and RhoA/ROCK. These signaling pathways contribute to anti-fibrosis, wound healing, anti-inflammatory, and protective effects across diverse non-malignant fibroblast models, whereas in cancer-associated fibroblasts, estrogen signaling exhibits context-dependent effects shaped by the tumor microenvironment. Together, these observations provide a consolidated view of estrogen-mediated fibroblast signaling and help contextualize divergent findings across physiological and pathological conditions.

## Supplementary material

Supplementary Table 1 Search Query strings for databases: **(a)**. PubMed, **(b)**. Scopus, and **(c)**. Embase.

Supplementary Table 2 Quality assessment of the included studies.
