# Supplementary material for: Estrogen signaling processes in fibroblasts: a scoping review
Source: Front Endocrinol (Lausanne). 2026 Mar 19;17:1768772. doi: 10.3389/fendo.2026.1768772 (PMC13044405; doi:10.3389/fendo.2026.1768772)
Supplement: Supplementary Table 1 — Search Query strings for databases: (a). PubMed, (b). Scopus, and (c). Embase. [file Table1.docx]

Supplementary Material

**Supplementary Table 1: Search query strings for databases: a) PubMed, b) Scopus, and c) Embase.**

1. **Query string for PubMed database**

|  | Query string | Results |
| --- | --- | --- |
| 1 | ((((((((fibroblasts[MeSH Terms]) OR (fibroblast*[Title/Abstract])) OR (vulval fibroblast*[Title/Abstract])) OR (dermal fibroblast*[Title/Abstract])) OR (vaginal fibroblast*[Title/Abstract])) OR (uterine fibroblast*[Title/Abstract])) OR (cardiac fibroblast*[Title/Abstract])) OR (placental fibroblast*[Title/Abstract])) OR (mammary fibroblast*[Title/Abstract]) | 377,879 |
| 2 | ((((((((((((((((((((((((((((((((((((((((((((((Estrogens[MeSH Terms]) OR (17 beta estradiol[MeSH Terms])) OR (estrogen receptor[MeSH Terms])) OR (G protein-coupled estrogen receptor 1[MeSH Terms])) OR (phytoestrogens[MeSH Terms])) OR (isoflavones[MeSH Terms])) OR (lignans[MeSH Terms])) OR (coumestrol[MeSH Terms])) OR (selective estrogen receptor modulators[MeSH Terms])) OR (tamoxifen[MeSH Terms])) OR (raloxifene[MeSH Terms])) OR (pathways, signal transduction[MeSH Terms])) OR (gene expression regulation[MeSH Terms])) OR (transcription, genetic[MeSH Terms])) OR (receptors, cytoplasmic and nuclear[MeSH Terms])) OR (extracellular signal regulated kinase[MeSH Terms])) OR (estrogen*[Title/Abstract])) OR (estradiol[Title/Abstract])) OR (estrogen receptor*[Title/Abstract])) OR (ERα[Title/Abstract])) OR (ERβ[Title/Abstract])) OR (GPER[Title/Abstract])) OR (GPR30[Title/Abstract])) OR (phytoestrogen*[Title/Abstract])) OR (genistein[Title/Abstract])) OR (daidzein[Title/Abstract])) OR (coumestrol[Title/Abstract])) OR (equol[Title/Abstract])) OR (lignan*[Title/Abstract])) OR (isoflavone*[Title/Abstract])) OR (SERM*[Title/Abstract])) OR (selective estrogen receptor modulator*[Title/Abstract])) OR (tamoxifen[Title/Abstract])) OR (raloxifene[Title/Abstract])) OR (signal* transduction[Title/Abstract])) OR (hormone signal*[Title/Abstract])) OR (MAP kinase*[Title/Abstract])) OR (gene expression[Title/Abstract])) OR (transcription[Title/Abstract])) OR (regulation[Title/Abstract])) OR (receptor activation[Title/Abstract])) OR (genomic signal*[Title/Abstract])) OR (extracellular signal-regulated kinase*[Title/Abstract])) OR (non-genomic signal*[Title/Abstract])) OR (cAMP[Title/Abstract])) OR (kinase cascade*[Title/Abstract])) OR (estrogen response element[Title/Abstract]) | 3,480,542 |
| 3 | ((((((((((((((((((in vitro techniques[MeSH Terms]) OR (cell line[MeSH Terms])) OR (cells, cultured[MeSH Terms])) OR (animals[MeSH Terms])) OR (humans[MeSH Terms])) OR (mice[MeSH Terms])) OR (rats[MeSH Terms])) OR (tissue culture techniques[MeSH Terms])) OR (models, biological[MeSH Terms])) OR (in vitro[Title/Abstract])) OR (in vivo[Title/Abstract])) OR (ex vivo[Title/Abstract])) OR (cultured cell*[Title/Abstract])) OR (cell line*[Title/Abstract])) OR (animal model*[Title/Abstract])) OR (human fibroblast*[Title/Abstract])) OR (primary fibroblast*[Title/Abstract])) OR (Mice*[Title/Abstract])) OR (rats*[Title/Abstract]) | 28,999,972 |
| 4 | 1 AND 2 AND 3 | 138,375 |
| 5 | #4 AND Filters applied: Comparative Study, Evaluation Study, Observational Study, Research Support, N.I.H., Extramural, Research Support, N.I.H., Intramural, Research Support, Non-U.S. Gov't, Research Support, U.S. Gov't, Non-P.H.S., Research Support, U.S. Gov't, P.H.S., Research Support, U.S. Gov't, Validation Study, English, Exclude preprints, from 2015/1 - 2026/7. | 26,004 |

1. **Query string for Scopus database**

|  | Query string | Results |
| --- | --- | --- |
| 1 | ( ( ( TITLE-ABS-KEY ( fibroblasts ) OR TITLE-ABS-KEY ( vulval fibroblast* ) OR TITLE-ABS-KEY ( dermal fibroblast* ) OR TITLE-ABS-KEY ( vaginal fibroblast* ) OR TITLE-ABS-KEY ( uterine fibroblast* ) OR TITLE-ABS-KEY ( cardiac fibroblast* ) OR TITLE-ABS-KEY ( placental fibroblast* ) OR TITLE-ABS-KEY ( mammary fibroblast* ) ) ) AND ( ( TITLE-ABS-KEY ( estrogen* ) OR TITLE-ABS-KEY ( estradiol ) OR TITLE-ABS-KEY ( estrogen receptor* ) OR TITLE-ABS-KEY ( ERα ) OR TITLE-ABS-KEY ( ERβ ) OR TITLE-ABS-KEY ( gper ) OR TITLE-ABS-KEY ( gpr30 ) OR TITLE-ABS-KEY ( phytoestrogen* ) OR TITLE-ABS-KEY ( genistein ) OR TITLE-ABS-KEY ( diadzein ) OR TITLE-ABS-KEY ( coumestrol ) OR TITLE-ABS-KEY ( equol ) OR TITLE-ABS-KEY ( lignan ) OR TITLE-ABS-KEY ( isoflavones ) OR TITLE-ABS-KEY ( SERM ) OR TITLE-ABS-KEY ( selective estrogen receptor modulator* ) OR TITLE-ABS-KEY ( tamoxifen ) OR TITLE-ABS-KEY ( raloxifene ) OR TITLE-ABS-KEY ( signal* transduction ) OR TITLE-ABS-KEY ( hormone signal* ) OR TITLE-ABS-KEY ( MAP kinase ) OR TITLE-ABS-KEY ( gene expression ) OR TITLE-ABS-KEY ( transcription ) OR TITLE-ABS-KEY ( regulation ) OR TITLE-ABS-KEY ( receptor activation ) OR TITLE-ABS-KEY ( genomic signal* ) OR TITLE-ABS-KEY ( extracellular signal-regulated kinase* ) OR TITLE-ABS-KEY ( non-genomic signal* ) OR TITLE-ABS-KEY ( cAMP ) OR TITLE-ABS-KEY ( kinase cascade ) OR TITLE-ABS-KEY ( estrogen response element ) ) ) AND ( ( TITLE-ABS-KEY ( invitro ) OR TITLE-ABS-KEY ( invivo ) OR TITLE-ABS-KEY ( exvivo ) OR TITLE-ABS-KEY ( cultured cell* ) OR TITLE-ABS-KEY ( cell line* ) OR TITLE-ABS-KEY ( animal model* ) OR TITLE-ABS-KEY ( human ) OR TITLE-ABS-KEY ( primary fibroblast* ) OR TITLE-ABS-KEY ( mice ) OR TITLE-ABS-KEY ( rats ) ) ) | 181,047 |
| 2 | AND PUBYEAR > 2014 AND PUBYEAR < 2026 ) AND ( fibroblasts ) AND ( estrogen ) AND ( LIMIT-TO ( DOCTYPE , "ar" ) ) AND ( LIMIT-TO ( EXACTKEYWORD , "Human" ) OR LIMIT-TO ( EXACTKEYWORD , "Fibroblast" ) OR LIMIT-TO ( EXACTKEYWORD , "Animals" ) OR LIMIT-TO ( EXACTKEYWORD , "Mouse" ) OR LIMIT-TO ( EXACTKEYWORD , "Male" ) OR LIMIT-TO ( EXACTKEYWORD , "Female" ) OR LIMIT-TO ( EXACTKEYWORD , "Signal Transduction" ) OR LIMIT-TO ( EXACTKEYWORD , "Animal Model" ) OR LIMIT-TO ( EXACTKEYWORD , "Fibroblasts" ) OR LIMIT-TO ( EXACTKEYWORD , "Mice" ) OR LIMIT-TO ( EXACTKEYWORD , "Upregulation" ) OR LIMIT-TO ( EXACTKEYWORD , "Western Blotting" ) OR LIMIT-TO ( EXACTKEYWORD , "Cell Culture" ) OR LIMIT-TO ( EXACTKEYWORD , "In Vitro Study" ) OR LIMIT-TO ( EXACTKEYWORD , "Real Time Polymerase Chain Reaction" ) OR LIMIT-TO ( EXACTKEYWORD , "Immunohistochemistry" ) OR LIMIT-TO ( EXACTKEYWORD , "Down Regulation" ) OR LIMIT-TO ( EXACTKEYWORD , "Cells, Cultured" ) OR LIMIT-TO ( EXACTKEYWORD , "In Vivo Study" ) OR LIMIT-TO ( EXACTKEYWORD , "Rat" ) OR LIMIT-TO ( EXACTKEYWORD , "Enzyme Linked Immunosorbent Assay" ) OR LIMIT-TO ( EXACTKEYWORD , "Reverse Transcription Polymerase Chain Reaction" ) OR LIMIT-TO ( EXACTKEYWORD , "Skin Fibroblast" ) OR LIMIT-TO ( EXACTKEYWORD , "Cell Proliferation" ) OR LIMIT-TO ( EXACTKEYWORD , "Gene Expression Regulation" ) OR LIMIT-TO ( EXACTKEYWORD , "Apoptosis" ) OR LIMIT-TO ( EXACTKEYWORD , "Estrogen Receptor" ) OR LIMIT-TO ( EXACTKEYWORD , "Cell Differentiation" ) OR LIMIT-TO ( EXACTKEYWORD , "Protein Phosphorylation" ) OR LIMIT-TO ( EXACTKEYWORD , "Immunofluorescence" ) OR LIMIT-TO ( EXACTKEYWORD , "Extracellular Matrix" ) OR LIMIT-TO ( EXACTKEYWORD , "Progesterone Receptor" ) OR LIMIT-TO ( EXACTKEYWORD , "Estrogen" ) OR LIMIT-TO ( EXACTKEYWORD , "Estrogen Receptor Alpha" ) OR LIMIT-TO ( EXACTKEYWORD , "Estradiol" ) OR LIMIT-TO ( EXACTKEYWORD , "Rats" ) OR LIMIT-TO ( EXACTKEYWORD , "Cell Survival" ) OR LIMIT-TO ( EXACTKEYWORD , "Fibrosis" ) ) AND ( LIMIT-TO ( LANGUAGE , "English" ) ) AND ( LIMIT-TO ( SRCTYPE , "j" ) ) AND ( LIMIT-TO ( SUBJAREA , "MEDI" ) OR LIMIT-TO ( SUBJAREA , "BIOC" ) OR LIMIT-TO ( SUBJAREA , "PHAR" ) ) | 4,329 |

1. **Query string for Embase database**

|  | Query | Results |
| --- | --- | --- |
| 1 | ('fibroblast')/br OR (('vulval fibroblast'):ti,ab,kw) OR (('uterine fibroblast'):ti,ab,kw) OR (('vaginal fibroblast'):ti,ab,kw) OR (('placental fibroblast'):ti,ab,kw) OR (('mammary fibroblast'):ti,ab,kw) OR (('cardiac fibroblast'):ti,ab,kw) OR (('dermal fibroblast'):ti,ab,kw) | 409,276 |
| 2 | ('hormone signaling')/br OR (('estrogen'):ti,ab,kw) OR (('estradiol'):ti,ab,kw) OR (('estrogen receptor'):ti,ab,kw) OR (('estrogen receptor alpha'):ti,ab,kw) OR (('estrogen receptor beta'):ti,ab,kw) OR (('G protein coupled receptor 30'):ti,ab,kw) OR (('phytoestrogen'):ti,ab,kw) OR (('genistein'):ti,ab,kw) OR (('daidzein'):ti,ab,kw) OR (('coumestrol'):ti,ab,kw) OR (('equol'):ti,ab,kw) OR (('lignan'):ti,ab,kw) OR (('isoflavone'):ti,ab,kw) OR (('selective estrogen receptor modulator'):ti,ab,kw) OR (('tamoxifen'):ti,ab,kw) OR (('raloxifene'):ti,ab,kw) OR (('signal transduction'):ti,ab,kw) OR (('hormone signaling'):ti,ab,kw) OR (('mitogen activated protein kinase'):ti,ab,kw) OR (('gene expression'):ti,ab,kw) OR (('transcription'):ti,ab,kw) OR (('regulatory mechanism'):ti,ab,kw) OR (('receptor activation'):ti,ab,kw) OR (('genomic signaling'):ti,ab,kw) OR (('non-genomic signaling'):ti,ab,kw) OR (('cyclic AMP'):ti,ab,kw) OR (('estrogen responsive element'):ti,ab,kw) | 1,882,803 |
| 3 | ('experimentation')/br OR (('in vitro study'):ti,ab,kw) OR (('in vivo study'):ti,ab,kw) OR (('ex vivo study'):ti,ab,kw) OR (('cell culture'):ti,ab,kw) OR (('cell line'):ti,ab,kw) OR (('animal model'):ti,ab,kw) OR (('human'):ti,ab,kw) OR (('mouse'):ti,ab,kw) OR (('rat'):ti,ab,kw) | 8,657,801 |
| 4 | 1 AND 2 AND 3 | 45,567 |
| 5 | #4 AND ('adrenergic receptor'/dd OR 'androgen'/dd OR 'androgen receptor'/dd OR 'antiandrogen'/dd OR 'antiestrogen'/dd OR 'aromatase'/dd OR 'bazedoxifene'/dd OR 'cell surface receptor'/dd OR 'cyclic amp'/dd OR 'cyclic amp responsive element binding protein'/dd OR 'estradiol'/dd OR 'estradiol 17beta dehydrogenase'/dd OR 'estradiol receptor'/dd OR 'estrogen'/dd OR 'estrogen receptor'/dd OR 'estrogen receptor alpha'/dd OR 'estrogen receptor alpha, human' OR 'estrogen receptor antagonist'/dd OR 'estrogen receptor beta'/dd OR 'estrogen related receptor alpha'/dd OR 'estrogen related receptor beta'/dd OR 'estrogen related receptor gamma'/dd OR 'estrone'/dd OR 'estrone sulfate'/dd OR 'fibroblast activation protein'/dd OR 'g protein coupled estrogen receptor'/dd OR 'g protein coupled receptor'/dd OR 'g protein coupled receptor kinase 5'/dd OR 'genistein'/dd OR 'hormone'/dd OR 'hormone receptor'/dd OR 'matrix metalloproteinase'/dd OR 'membrane receptor'/dd OR 'mitogen activated protein kinase'/dd OR 'mitogen activated protein kinase kinase'/dd OR 'mitogen activated protein kinase kinase 1'/dd OR 'peroxisome proliferator activated receptor gamma coactivator 1alpha'/dd OR 'phospholipase c'/dd OR 'phytoestrogen'/dd OR 'platelet derived growth factor alpha receptor'/dd OR 'platelet derived growth factor beta receptor'/dd OR 'progesterone'/dd OR 'progesterone receptor'/dd OR 'progesterone receptor a'/dd OR 'progesterone receptor b'/dd OR 'protein kinase'/dd OR 'protein tyrosine kinase'/dd OR 'raloxifene'/dd OR 'ras protein'/dd OR 'sex hormone'/dd OR 'steroid hormone'/dd OR 'steroid receptor'/dd OR 'steroidogenic acute regulatory protein'/dd OR 'sterol regulatory element binding protein 1'/dd OR 'sterol regulatory element binding protein 1c'/dd OR 'tamoxifen'/dd) | 2,471 |
