# Supplementary material for: Estrogen signaling processes in fibroblasts: a scoping review
Source: Front Endocrinol (Lausanne). 2026 Mar 19;17:1768772. doi: 10.3389/fendo.2026.1768772 (PMC13044405; doi:10.3389/fendo.2026.1768772)
Supplement: Supplementary Table 2 — Quality assessment of the included studies. [file Table2.docx]

Supplementary Material

**Supplementary Table 2: Quality assessment of the included studies**

| **Author Name/ year** | **Journal** | **H5-Index** | **2024 journal Impact factor** | **Scimago Journal Rank 2024** |
| --- | --- | --- | --- | --- |
| Aguado et al., 2020 | Cells | 174 | 5.2 | 1.670 Q1 |
| Ahluwalia et al., 2022 | Endocrinology | 54 | 3.3 | 1.308 Q1 |
| Almuntashiri et al., 2024 | Physiological Reports | 43 | 1.9 | 0.766 Q2 |
| Avouac et al., 2020 | Journal of Investigative Dermatology | 71 | 5.7 | 1.659 Q1 |
| Bae et al., 2022 | The FASEB Journal | 86 | 4.2 | 1.319 Q1 |
| BakerFrost et al., 2024 | International Journal of Molecular Sciences | 277 | 4.9 | 1.273 Q1 |
| BakerFrost et al., 2021 | Arthritis Research & Therapy | 56 | 4.6 | 1.587 Q1 |
| Carnesecchi et al., 2015 | PLOS ONE | 244 | 2.6 | 0.803 Q1 |
| Cheng et al., 2025 | Biocell | 18 | 1 | 0.252 Q4 |
| Darawsha et al., 2021 | Antioxidants | 154 | 6.6 | 1.484 Q1 |
| Darawsha et al., 2024 | Antioxidants | 154 | 6.6 | 1.484 Q1 |
| DeMarco et al., 2016 | Scientific Reports | 234 | 3.9 | 0.874 Q1 |
| Dworatzek et al., 2019 | Cardiovascular Research | 100 | 13.3 | 3.947 Q1 |
| Feng et al., 2019 | Journal of Oral and Maxillofacial Surgery | 41 | 2.6 | 0.767 Q1 |
| He et al., 2024 | Clinical and Translational Medicine | 71 | 6.8 | 3.030 Q1 |
| Hu et al., 2024 | Nature Communications | 399 | 15.7 | 4.761 Q1 |
| Jia et al., 2016 | Endocrinology | 54 | 3.3 | 1.308 Q1 |
| Jiang et al., 2015 | International Journal of Molecular Medicine | 62 | 5.8 | 1.381 Q1 |
| Kaňuchová et al., 2021 | Physiological Research | 36 | 2 | 0.676 Q2 |
| Kim et al., 2022 | Stem Cell Research & Therapy | 99 | 7.3 | 2.021 Q1 |
| Kwon et al., 2024 | BMB Reports |  | 3.3 | 1.125 Q1 |
| Leite et al., 2023 | Brazilian Journal of Medical and Biological Research | 31 | 1.5 | 0.498 Q2 |
| LiebenLouis et al., 2019 | Molecules | 203 | 4.6 | 0.865 Q1 |
| Ling et al., 2025 | Inflammatory Bowel Diseases | 61 | 4.3 | 1.780 Q1 |
| Liu et al., 2022 | Molecular Biology Reports | 66 | 2.8 | 0.710 Q2 |
| Liu et al., 2021 | Aging | 94 | 3.9 | 1.078 Q2 |
| Lu et al., 2024 | Genes & Nutrition |  | 4.9 | 1.008 Q2 |
| Luo et al., 2016 | Steroids | 31 | 2.3 | 0.620 Q2 |
| Ma et al., 2021 | American journal of translational research | 46 | 1.6 | Discontinued in Scopus as of 2021 |
| Maggiolini et al., 2015 | Future Medicinal Chemistry | 41 | 3.4 | 0.501 Q3 |
| Malik et al., 2024 | Metabolites | 82 | 3.7 | 0.996 Q2 |
| Midgley et al., 2016 | Aging Cell | 84 | 7.1 | 2.905 Q1 |
| Morgan et al., 2018 | Scientific Reports | 234 | 3.9 | 0.874 Q1 |
| Nanashima et al., 2018 | Nutrients | 213 | 5 | 1.473 Q1 |
| Ouyang et al., 2016 | Scientific Reports | 234 | 3.9 | 0.874 Q1 |
| Ozawa et al., 2021 | The Laryngoscope | 60 | 2 | 1.029 Q1 |
| Patel et al., 2018 | American Journal of Reproductive Immunology | 40 | 2.4 | 0.835 Q2 |
| Patel et al., 2021 | Frontiers in Immunology | 224 | 5.9 | 1.941 Q1 |
| Patel et al., 2018 | Journal of Interferon & Cytokine Research | 20 | 1.8 | 0.628 Q2 |
| Pedram et al., 2016 | Molecular and Cellular Endocrinology | 55 | 3.6 | 1.198 Q1 |
| Pomari et al., 2015 | The FASEB Journal | 86 | 4.2 | 1.319 Q1 |
| Qin et al., 2015 | British Journal of Pharmacology | 92 | 7.7 | 2.344 Q1 |
| Santolla et al., 2015 | Cell Death & Disease | 136 | 9.6 | 2.773 Q1 |
| Santos et al., 2017 | Biology of Sex Differences | 45 | 5.1 | 1.799 Q1 |
| Savoia et al., 2018 | Journal of Dermatological Science | 34 | 4 | 1.082 Q1 |
| Shin et al., 2017 | International Journal of Molecular Sciences | 277 | 4.9 | 1.273 Q1 |
| Song et al., 2019 | PLOS ONE | 244 | 2.6 | 0.803 Q1 |
| Turczyk et al., 2017 | Neoplasia | 41 | 7.7 | 2.167 Q1 |
| Vivacqua et al., 2015 | Oncotarget | 41 | 5.168 | 0.785 Q2 |
| Vivacqua et al., 2018 | Cells | 174 | 5.2 | 1.670 Q1 |
| Wang et al., 2025 | Journal of Steroid Biochemistry and Molecular Biology | 43 | 2.5 | 0.729 Q2 |
| Wang et al., 2018 | DNA and Cell Biology | 33 | 2.6 | 0.637 Q2 |
| Wang et al., 2020 | Frontiers in Endocrinology | 127 | 4.6 | 1.472 Q1 |
| Wang et al., 2021 | Frontiers in Pharmacology | 145 | 4.8 | 1.220 Q1 |
| Wang et al., 2019 | Life Sciences | 108 | 5.1 | 1.315 Q1 |
| Wang et al., 2015 | Molecular and Cellular Biochemistry | 54 | 3.7 | 1.033 Q1 |
| Wang et al., 2018 | Biochemical and Biophysical Research Communications | 76 | 2.2 | 0.748 Q2 |
| Wu et al., 2017 | Development | 67 | 3.6 | 1.715 Q1 |
| Xie et al., 2022 | Frontiers in Physiology | 112 | 3.4 | 1.023 Q2 |
| Xu et al., 2024 | Molecular Medicine Reports | 56 | 3.5 | 0.841 Q2 |
| Yamanaka et al., 2018 | Journal of Cellular Physiology | 86 | 4 | 1.377 Q1 |
| Yang et al., 2023 | International Journal of Ophthalmology | 29 | 1.8 | 0.544 Q2 |
| Yu et al., 2017 | Oncogene | 93 | 7.3 | 2.489 Q1 |
| Yuan et al., 2015 | Breast Cancer Research | 55 | 5.6 | 2.202 Q1 |
| Zhang et al., 2023 | Journal of Cosmetic Dermatology | 59 | 2.5 | 0.764 Q2 |
| Zhang et al., 2018 | American journal of translational research | 46 | 1.6 | Discontinued in Scopus as of 2021 |
| Zhao et al., 2015 | Australasian Journal of Dermatology | 31 | 1.8 | 0.581 Q2 |
